# Spatiotemporal dynamics of giant viruses within a deep freshwater lake reveal a distinct dark-water community

**DOI:** 10.1093/ismejo/wrae182

**Published:** 2024-09-23

**Authors:** Liwen Zhang, Lingjie Meng, Yue Fang, Hiroyuki Ogata, Yusuke Okazaki

**Affiliations:** Bioinformatics Center, Institute for Chemical Research, Kyoto University, Uji, Kyoto 611-0011, Japan; Bioinformatics Center, Institute for Chemical Research, Kyoto University, Uji, Kyoto 611-0011, Japan; Bioinformatics Center, Institute for Chemical Research, Kyoto University, Uji, Kyoto 611-0011, Japan; Bioinformatics Center, Institute for Chemical Research, Kyoto University, Uji, Kyoto 611-0011, Japan; Bioinformatics Center, Institute for Chemical Research, Kyoto University, Uji, Kyoto 611-0011, Japan

**Keywords:** viral ecology, giant viruses, Nucleocytoviricota, Mirusviricota, deep freshwater lakes

## Abstract

Giant viruses (GVs) significantly regulate the ecological dynamics of diverse ecosystems. Although metagenomics has expanded our understanding of their diversity and ecological roles played in marine environments, little is known about GVs of freshwater ecosystems. Most previous studies have employed short-read sequencing and therefore resulted in fragmented genomes, hampering accurate assessment of genetic diversity. We sought to bridge this knowledge gap and overcome previous technical limitations. We subjected spatiotemporal (2 depths × 12 months) samples from Lake Biwa to metagenome–assembled genome reconstruction enhanced by long-read metagenomics. This yielded 293 GV metagenome-assembled genomes. Of these, 285 included previously unknown species in five orders of nucleocytoviruses and the first representatives of freshwater mirusviruses, which exhibited marked divergence from marine-derived lineages. The good performance of our long-read metagenomic assembly was demonstrated by the detection of 42 (14.3%) genomes composed of single contigs with completeness values >90%. GVs were partitioned across water depths, with most species specific to either the sunlit epilimnion or the dark hypolimnion. Epilimnion-specific members tended to be transient and exhibit short and intense abundance peaks, in line with the fact that they regulate the surface algal blooms. During the spring bloom, mirusviruses and members of three nucleocytovirus families were among the most abundant viruses. In contrast, hypolimnion-specific ones, including a mirusvirus genome, were typically more persistent in the hypolimnion throughout the water-stratified period, suggesting that they infect hosts specific to the hypolimnion and play previously unexplored ecological roles in dark water microbial ecosystems.

## Introduction

Giant viruses (GVs) are a significant group within the virosphere, exhibiting remarkable diversity, ubiquity, and abundance across various ecosystems such as oceans, freshwater, and soil [1–6]. In marine ecosystems, they are widespread and distributed across the water column. In contrast, the diversity of freshwater GVs has not been well studied despite indications of high diversity, as revealed by the distribution of their major capsid proteins (MCPs) in freshwater environments [4].

Freshwater lakes, known for their complex seasonal and vertical dynamics, have been the subject of extensive studies in terms of the temporal shifts and vertical stratification of plankton and prokaryote communities [7–10]. These studies have revealed the niche preferences of eukaryotic and prokaryotic microbes across seasons and depths, highlighting the existence of a deep-water specific microbiome. A recent study revealed the dominant GVs associated with spring algal blooms in photic zones [11]. However, the ecological dynamics of GVs in freshwater lakes remain poorly understood and no study has previously addressed the existence of GVs specific to the dark and deep layers of a lake.

We comprehensively analyzed the diversity of GVs in a deep freshwater ecosystem via reconstruction of metagenome-assembled genomes (MAGs) for investigating the GV dynamics across seasons and depths. To achieve this, we combined a spatiotemporal sampling strategy with long-read metagenomic sequencing. This enabled us to capture the GV community dynamics within the ecosystem and overcame the problem posed by fragmented assembly of conventional short-read metagenomes [12]. The generation of more continuous contigs via long-read sequencing aids the identification of a full set of marker genes for MAGs, allowing more accurate quality evaluation and taxonomic assignment. Indeed, an increasing number of studies have used long-read sequencing to generate better GV genomes [13–15], but no long-read GV MAG has been generated from freshwater metagenomic data. Moreover, we developed a pipeline that detected not only GVs of the phylum *Nucleocytoviricota* but also those of *Mirusviricota*, a newly discovered GV phylum [5]. Previous metagenomic studies have often overlooked mirusviruses given their high genomic novelty and chimeric attributes [5]. Indeed, mirusviruses in the freshwater ecosystems remain to be discovered.

We leveraged previously published short- and long-read metagenomic data from Lake Biwa, a deep oligo-mesotrophic freshwater lake of Japan [16]. The data originally targeted the prokaryotic community (size fraction = 0.2–5 μm) and were collected spatiotemporally. GVs have the same size fraction as prokaryotes [17] but were not investigated in the original study. We reanalyzed these data using a custom pipeline that recovers GV genomes from long-read contigs and bins. This led to the reconstruction of 118 high-quality (completeness >90%) GV MAGs, including two circular nucleocytoviruses and eight representatives of freshwater mirusviruses. Moreover, the spatiotemporal data revealed the dynamics of GV communities and their specific occurrences across the depths.

## Materials and methods

### Data source

We compiled long-read MAGs and contigs derived in a recent study on Lake Biwa, Japan [16]. Dataset samples were collected monthly from May 2018 to April 2019. Throughout the 1-year sampling period, thermal stratification occurred from May to December. During each sampling event, water samples from two depths (5 m for the epilimnion and 65 m for the hypolimnion) were collected (24 samples in total). Deoxyribonucleic acid (DNA) was extracted from the 0.22–5 μm size fraction of each sample and subjected to short-read (MGI DNBSEQ-G400) and long-read metagenomic sequencing (Oxford Nanopore). The contigs used here were those assembled by Flye v2.8 [18] in the previous study. The long-read contigs were polished using both the long and short reads. The detailed workflow and the relevant parameters are described in the original publication [16].

### Reconstruction of long-read giant virus metagenome-assembled genomes

Many contigs exceeded the minimum size criterion for a GV genome (>50 kb) and displayed GV signals (≥1/7 nucleocytovirus marker genes or the mirusvirus HK97 MCP). The seven nucleocytovirus marker genes were those encoding MCP, DNA polymerase family B (PolB), transcription initiation factor IIB (TFIIB), DNA topoisomerase II (TopoII), packaging ATPase (A32), DEAD/SNF2-like helicase (SFII), and the poxvirus late transcription factor VLTF3 [19]. In addition to these contigs, we retained all 4648 bins generated in the original study [16], which together subjected to the exclusion of prokaryotic genomes with CheckM v1.2.2 [20]. MAGs with CheckM completeness scores higher than 15 as bacteria or 20 as archaea were considered to be prokaryotes and therefore excluded (Fig. S1A). Additionally, we reevaluated bins excluded by this process and confirmed that there was no significant loss of high-quality GV sequences due to the additional recruitment of single-contig GVs in parallel with binned data (Fig. S1B; see the Supplementary methods).

We screened for putative GV MAGs using different methods (see Supplementary methods) to identify nucleocytoviruses and mirusviruses. For nucleocytoviruses, we employed a core gene density index based on the presence of 20 nucleocytovirus core genes to select putative nucleocytovirus MAGs for further examinations [21]. To detect mirusviruses, we screened for the mirusvirus HK97 MCP gene as this is a unique marker of mirusviruses [5]. A MAG was identified as a mirusvirus if the HK97 MCP gene was detected using the function “hmmsearch” of HMMER3 v3.4 (bit score > 100) [22].

Following the MAG detection, we removed all cellular contamination and then excluded chimeric, low-quality, and fragmented GV MAGs prior to downstream analyses (Figs S2 and S3; see the Supplementary methods). Finally, 293 non-redundant GV MAGs generated by the above processes were retained at an average nucleotide identity (ANI) threshold of 95% with dRep v3.2.2 [23]. The resulting non-redundant GV MAGs were species-level representatives that we termed “Lake Biwa giant virus metagenome-assembled genomes” (LBGVMAGs). Each was assigned a unique four-digit serial number as part of the ID, ordered by the maximum coverage rank across all 24 samples.

### Quality assessment of long-read giant virus metagenome-assembled genomes

We first assessed the diversity-coverage of our MAGs by determining the proportion of uncaptured nucleocytovirus *polB* sequences. The unique nature of this gene, which is single-copy and universal in nucleocytovirus genomes, allowed us to assess how much of the GV diversity in the lake was captured by our MAGs. We performed a blastn search using blast+ v2.15.0 [24] to align all representatives of clustered *polB* sequences from the raw assemblies (see the Supplementary methods) against the contigs of our MAGs. A *polB* sequence was considered to be present in our GV MAGs if the nucleotide sequence identity was >96% and aligned length covered >60% of the shorter sequence in a pair. These two thresholds were estimated to roughly represent the species boundary of GVs (Fig. S4; see the Supplementary methods).

To compare the fragmentation levels of our long-read MAGs and those of the short-read MAGs, we compiled non-redundant quality-controlled (high/medium quality) short-read GV MAGs from the Giant Virus Database (GVDB) [19]. Seqkit v2.5.1 [25] was employed to calculate the number of contigs and the N50 value of each MAG. We also determined the POA90 score of each MAG; this metric evaluates unpolished indel errors in long-read assemblies [16]. The details of quality assessment are given in the Supplementary methods.

### Analyses of the phylogenetic diversity and community dynamics of GV MAGs

To evaluate the novelty of our MAGs, we complied a custom database that integrated the GVDB [19] and 697 nucleocytovirus/mirusvirus MAGs recovered from “Tara Oceans” and lodged in the Global Ocean Eukaryotic Viral database [5]. This custom database followed the taxonomic classification of the GVDB. Next, we used fastANI v1.33 with the default parameters to calculate the pairwise ANIs between this custom database and our MAGs [26]. The alignments were visualized with DiGAlign [27].

For phylogenetic analysis of the nucleocytoviruses, we used the “ncldv_markersearch.py” script to call seven marker genes (encoding PolB, SFII, TFIIB, TopoII, A32, VLTF3, and the DNA-directed ribonucleic acid polymerase alpha subunit [RNAPL]) [3] from our MAGs and reference genomes. We then generated a concatenated alignment of the seven genes using MAFFT v7.520 [28] with the “L-INS-i” algorithm and trimmed the alignment at >90% gaps using trimAl [29]. We generated a phylogenetic tree with IQ-TREE v2.2.2.6 [30] using Ultrafast Bootstrap [31] (parameters: -wbt -bb 1000) and visualized the tree using iTOL [32]. The best-fitting model (LG + F + I + R10) was selected according to the Bayesian information criterion from the ModelFinder [33]. The taxonomy of our MAGs was manually determined based on the topology of the tree following the taxonomic classification of the GVDB.

To infer mirusvirus phylogeny, we generated individual phylogenetic trees of HK97 MCP and heliorhodopsin (HeR) sequences. We searched against an Hidden Markov Model (HMM) model built from marine mirusviruses [5] to screen for HK97 MCPs using the hmmsearch (bit score > 100), and another HMM model generated from the custom database described above to screen for HeRs with an E-value of 1 × 10^−3^. For both phylogenetic trees, we included sequences from our MAGs and marine mirusviruses. Additionally, we incorporated reference sequences from recently identified endogenous mirusviruses in the HK97 MCP tree [34] and sequences from the RefSeq database [35] for the HeR tree. We excluded sequences of the marine mirusvirus family M7 from both trees due to the phylogenetic instability introduced by them. Alignments of the HK97 MCPs and HeRs were generated using MAFFT v7.520 with the “L-INS-i” algorithm and trimmed at gaps >90% with trimAl v1.4. Trees were built using IQ-TREE v2.2.2.6 with the Ultrafast Bootstrap parameters “-B 1000 -alrt 1000”. Model “LG + F + R7” and “VT + F + R8” were selected to generate the trees of HK97 MCPs and HeRs, respectively. Phylogenetic inferences aside, we further quantified the shared genomic content among freshwater mirusviruses, marine mirusviruses, and other members of the realm *Duplodnaviria* from Virus–Host Database [36], as detailed in the Supplementary methods.

To determine the relative abundances and spatiotemporal distributions of LBGVMAGs, the coverage and read per kilobase per million reads (RPKM) of each MAG were calculated based on the mapping of short reads from all samples to LBGVMAGs using CoverM v0.6.1 [37] with parameters “--min-read-percent-identity 0.92 -p bwa-mem2”. The taxonomic composition was based on the RPKMs of each order per sample and visualized using the ggplot2 package [38] in R Studio [39, 40]. The beta diversity between different communities was calculated using the vegdist function (method = “bray”) of the vegan package [41] and used for nonmetric multidimensional scaling (NMDS) analysis. The quantitative analysis that compared the compositional variances between the epilimnion and hypolimnion used the same distance matrix, but we limited our analysis to samples collected during the period of water stratification (May–December). The abundance profile of plankton in Lake Biwa at sampling months were downloaded from a publicly available plankton monitoring project [42].

The habitat preference of LBGVMAGs was assessed by an indicator termed “P_epi_”, which is the cumulative RPKM in the epilimnion divided by the cumulative RPKM in both epilimnion and hypolimnion during the stratification period (May–December) [16]. When P_epi_ was > 0.95 or < 0.05, the LBGVMAG was defined as epilimnion- or hypolimnion-specific, respectively.

The persistence of each LBGVMAG was defined as the longest consecutive months during the stratified period for which the covered fraction of the LBGVMAG was >20% from short-read mapping [43]. Persistence of epilimnion-specific LBGVMAGs was assessed using only epilimnion samples and persistence of hypolimnion-specific LBGVMAGs using hypolimnion samples. Statistical test of differences in the mean persistence between the two groups employed the Welch *t*-test.

## Results

### High quality long-read assembled giant virus metagenome-assembled genomes

**Figure 1:**
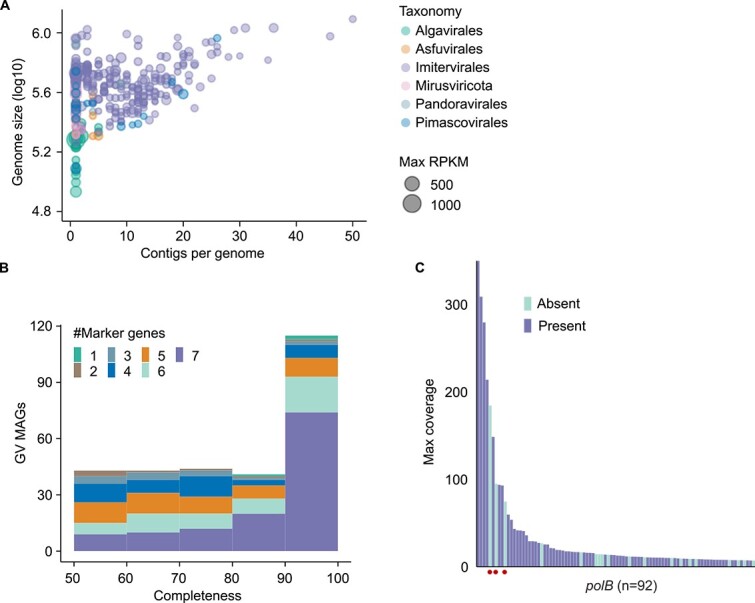
GV MAG quality and diversity coverage. (A) Genome size of GV MAGs versus the number of contigs. The size of each bubble represents the maximum RPKMs of each MAG in all 24 samples. (B) Distribution of the GV MAG completeness scores. Marker genes indicated here are nucleocytovirus marker genes. (C) Presence or absence of abundant *polB* genes in the GV MAGs. “Max coverage” refers to the highest read coverage of the contig from which the *polB* was called among the 24 samples. Dots indicate the three abundant *polB* genes absent from our MAGs.

Within the 24 samples, 0.5%–4.2% (mean = 2.1%) of the short reads were mapped onto the LBGVMAGs (hereafter, GV MAGs or MAGs when there is no ambiguity). The percentage of mapped reads was typically larger for the epilimnion than the hypolimnion (Fig. S5). Using these samples, we successfully reconstructed 293 non-redundant species-level GV MAGs (Table S1). The assembly became more challenging with increasing genome size, typically resulting in a higher number of contigs (Fig. 1A). Despite these challenges, the high quality of our GV MAGs was verified by comparison with previously reported short-read MAGs, and via completeness assessment. Our long-read MAGs demonstrated a significantly (*P* value = 6.8 × 10^−6^) lower median number of contigs, with a count of 6 compared to 10 for short-read MAGs, and the median N50 of long-read contigs was also significantly (*P* value = 8.4 × 10^−16^) longer than short-read MAGs by threefold (Fig. S6). We obtained 118 (40.3%) GV MAGs with completeness scores >90% that were classified as “high-quality” [44], of which 74 (62.7%) MAGs contained all seven marker genes (Fig. 1B). Among the 118 high-quality MAGs, 42 were composed of single contig, including 2 MAGs (0074 and 0028) previously identified as circular [16]. Additionally, we identified terminal inverted repeats in six single-contig MAGs, which are the signature of linear complete genomes (Table S2).

A large proportion of the *polB* genes (79.3%) in the assembled contigs were present in our GV MAGs, indicating that our MAGs represented most of the GV diversity present in the lake (Fig. 1C). Upon closer examination, the three most abundant nucleocytovirus *polB* genes absent from the MAGs were encoded in cellular contigs, suggesting that our pipeline effectively eliminated contamination of cellular sequences. The POA90 score that assessed the performance of contig polishing (see the Supplementary methods) decreased when the short-read coverage was below 7× (Fig. S7), suggesting that a short-read coverage >7× was required for effective nucleotide error correction.

### Expanded diversity of giant viruses

**Figure 2:**
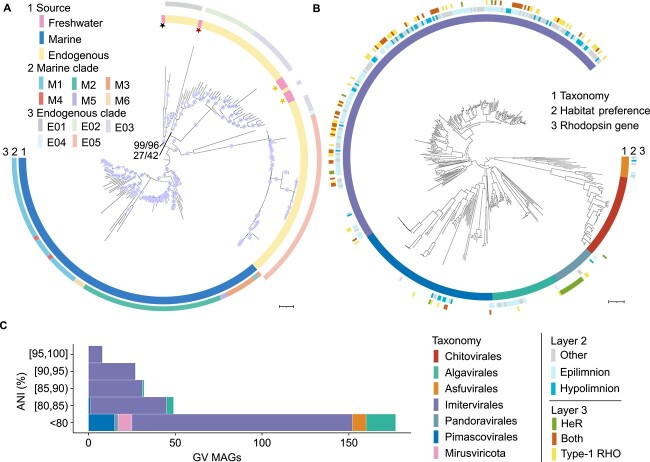
Phylogenetic diversity and novelty of GV MAGs. (A) Phylogenetic tree of the mirusvirus HK97 MCPs. Sequences from marine mirusvirus MAGs [5], endogenous mirusvirus sequences [43], and freshwater mirusvirus MAGs recovered in this study are included. The ultrafast bootstrap values are indicated by the sizes of the circles on the branches. Certain key nodes indicating the divergences of different clades are indicated by values (aLRT/UFBoot) (see Materials and methods). Phylogenetic supports were considered high (aLRT ≥80 and UFBoot ≥95), medium (aLRT ≥80 or UFBoot ≥95), or low (aLRT <80 and UFBoot <95). The three proposed subclades of freshwater mirusviruses are marked with stars on the tree in a clockwise direction: subclade2 (black), subclade1 (red), and two clusters of subclade3 (yellow). The tree is rooted between the freshwater and marine clades. Scale bar represents one substitution per site. (B) Phylogenetic tree of the recovered nucleocytoviruses. The tree was built using the concatenated protein sequences of seven genes (PolB, TFIIB, TopoII, A32, SFII, VLTF3, and RNAPL) and is rooted between the class *Pokkesviricetes* and *Megaviricetes*. The first outer layer of the tree (from the inside) indicates the taxonomic order of each GV genome, including our GV MAGs and the reference GV genomes. In the second layer, the uncolored branches of the tree are the reference genomes used to guide taxonomic assignment of our GV MAGs. “Other” means that the habitat preference was unclear. The third layer indicates the presence or absence of type-1 rhodopsin (Type-1 RHO)/HeR-encoding genes in our GV MAGs and the reference genomes. Scale bar represents one substitution per site. (C) The highest ANI value for each of our GV MAGs compared to the public GV genomes. Pairwise ANIs were calculated between our MAGs and publicly available GV genomes and only the highest ANI for each MAG is plotted. ANIs <80% are not specified, being rather clustered into the “<80” category.

The GV MAGs included mirusviruses (Fig. 2A) and five orders of nucleocytoviruses (Fig. 2B). We identified 285 new species that did not show >95% ANI with any known GV genome (Fig. 2C). Among them, 177 (60.4%) had ANI values <80% or ANI values that could not be calculated because of large divergences from the reference genomes. Of interest, 85.9% of GV MAGs with assigned ANIs were closely related to genomes from freshwater environments (Table S3). We also discovered three GV MAGs of the *Mesomimiviridae* (IM_01) family that were nearly identical (Fig. S8) to the MAGs recovered from Lake Lanier in North America [3, 4]. Specifically, MAGs 0129, 0046, and 0059 exhibited ANIs of 99.2%, 98.2%, and 98.2% to the MAGs from Lake Lanier, respectively. The order *Imitervirales* exhibited pronounced diversity, accounting for 237 (80.9%) MAGs in this study. Also, we recovered 20 *Algavirales*, 16 *Pimascovirales*, 8 *Asfuvirales*, and 4 *Pandoravirales*.

Following the screening approach guided by the mirusvirus HK97 MCP gene, we recovered eight mirusviruses from Lake Biwa, including the abovementioned circular genome (0074). HK97 MCP aside, other key components of the virion module were also shared with marine mirusviruses, including the genes encoding the portal protein, terminase, and the triplex capsid protein (Fig. S9A). The phylogenetic trees inferred based on HK97 MCPs (Fig. 2A) and HeRs (Fig. S10) supported a distant evolutionary relationship between the newly identified freshwater clade and known marine mirusviruses. For the freshwater clade, we further subdivided the group into three subclades based on the topology of the HK97 MCP tree (subclade1: 0074; subclade2: 0010; subclade3: the remaining six members). To date, the taxonomic classification of mirusviruses has been following the topology of HK97 MCP tree [5, 34] because it is considered as the key gene to guide the taxonomic classification of *Duplodnaviria*. Subclade3 was placed within the clade E03 of endogenous mirusviruses identified from two assemblies of algae under the class *Cryptophyceae* [34]. Subclade1 and subclade2 were placed next to the deep branches of endogenous mirusviruses clade E02 and E01, respectively (Fig. 2A). Consistent with the phylogenetic inferences suggested by the HK97 MCPs, the GC content of freshwater mirusviruses was higher than that of marine mirusvirus clades (Fig. S9B). Further genomic analysis of this freshwater clade revealed that a small fraction of genes (4.0%–10.8%) per genome were detected to be homologous to those of the marine mirusvirus orthogroups (Fig. S11; Table S4). Although limited, this genomic similarity was greater than that observed with other members of the realm *Duplodnaviria* (i.e. caudoviruses and herpesviruses; 0.6%–1.9%).

### Depth–dependent distribution patterns and functional capacities of giant viruses

**Figure 3:**
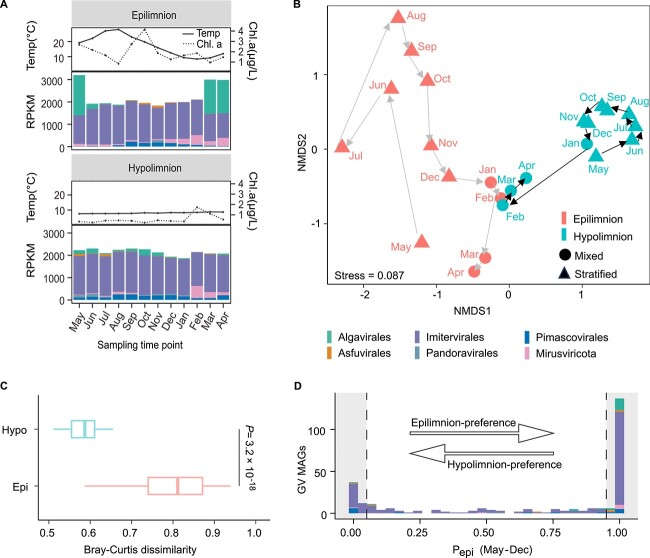
Spatiotemporal community shifts and vertical distributions. (A) Accumulative RPKM values across five orders of nucleocytoviruses and mirusviruses per sample. Within each bar, the abundance contributions made by each order or mirusvirus are indicated by different colors. The environmental parameters were also indicated by the dashed line (chlorophyll a) and solid line (temperature). (B) NMDS plot of beta diversities across the samples. The pairwise beta diversities were derived by calculating Bray–Curtis dissimilarities based on the RPKMs from read mapping. Triangles refer to samples from the stratification period. Circles represent samples taken during water mixing. (C) Comparison of the beta diversities of samples from the epilimnion and hypolimnion. The Bray–Curtis dissimilarities indicate the beta diversities for each pair of samples in the epilimnion or hypolimnion. (D) Habitat preference of each MAG as determined by the indicator P_epi_. As indicated by the dashed lines, MAGs with P_epi_ values >0.95 and < 0.05 were defined as epilimnion- and hypolimnion-specific, respectively.

The order-level community compositions and beta diversities among samples clearly revealed the succession of GV communities over the year (Fig. 3). After the water stratified (May–December), water circulation commenced and the GV communities of different water layers became well-mixed in February. A drastic shift in the hypolimnion community was apparent from January to February; however, the year-round cycle of the epilimnion community evidenced a more gradual change. This phenomenon is consistent with the mixing mechanism of lake water. As the temperature drops, the boundary of mixing water begins to gradually descend, and previously unmixed water is thus added to the epilimnion. In contrast, the hypolimnion is not affected until the mixing boundary attains the sampling depth (65 m). Throughout the stratification period, the compositional variation among communities in the hypolimnion was generally smaller than in the epilimnion (Fig. 3C). In line with NMDS analyses, most GV species (65.5%) were niche-specific. Specifically, 143 (48.8%) were epilimnion-specific and 49 (16.7%) were hypolimnion-specific (Fig. 3D).

Investigations on the coding capability of GV MAGs has revealed a rich diversity of metabolic genes that suggested possible adaptations at depths (Table S5). In addition to the most widely identified KEGG Orthology (KO) groups [45] involved in genetic information processing, those involved in glycan, nucleotide, amino acid, and carbohydrate metabolism were also prevalent (Fig. S12A). In total, we identified 1518 KOs from our GV MAGs. Of these, around half (656) were shared among GV MAGs with habitat preferences and those without such preferences (Fig. S12B), indicating their broad ecological relevance. We also identified 176 and 27 KOs exclusive to epilimnion- and hypolimnion-specific GV MAGs, respectively (Fig. S12B). The distinct distributions of KOs across depths suggest distinct metabolic adaptations at depths. For example, carotenoid cleavage oxygenase was detected to be unique to epilimnion-specific MAGs, indicating a possible adaptation to higher light exposure and oxidative conditions through manipulation of host carotenoid biosynthesis pathways. Despite these possible adaptations, the absence of highly prevalent habitat-specific genes (Fig. S12C) suggests a genetic versatility in their adaptation to environments.

Besides to the assigned KOs, we also widely detected GV rhodopsins (HeRs and type-1 rhodopsins). Specifically, 15 (30.6%) hypolimnion-specific and 57 (38.5%) epilimnion-specific GV species harbored rhodopsin genes (Fig. S13). HeR genes were present in most freshwater mirusvirus MAGs (7/8). In contrast, rhodopsins were least commonly observed in the order *Algavirales*.

### Distinct dynamics of epilimnion and hypolimnion specialists

**Figure 4:**
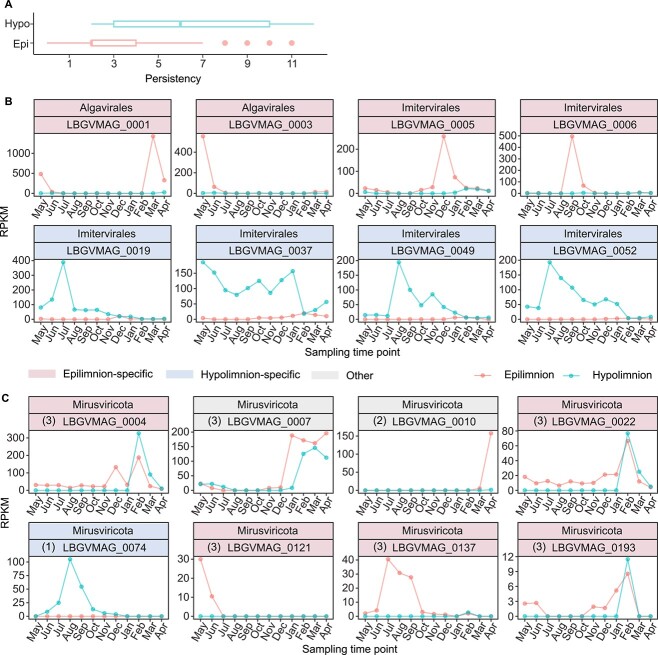
Community dynamics of GVs across water depths. (A) Persistence of GVs with different habitat preferences. Epi, epilimnion community; hypo, hypolimnion community. (B) Community dynamics of the top four most-abundant epilimnion- and hypolimnion-specific nucleocytoviruses (determined by the maximum RPKMs across the samples). (C) Community dynamics of freshwater mirusviruses. Subclades of freshwater mirusviruses are indicated by the numbers in parentheses followed by the MAG IDs. In (B) and (C), the taxonomy and ID of each MAG are shown in the title of each box, and the background indicates the habitat preference.

In general, GVs exhibiting different habitat preferences evidenced distinctive dynamic patterns. Epilimnion-specific GVs were typically transient but hypolimnion specialists tended to be more persistent as indicated by their significantly higher persistence (*P* value = 6.7 × 10^−6^) during the stratification period (Figs 4, S14, and S15). The median persistence duration for hypolimnion-specific GVs was 6 months, which is 3-fold longer than the 2-month median observed for epilimnion-specific GVs.

Overall, imiterviruses dominated both water layers throughout the year, with the exception that four algaviruses collectively accounted for >50% of the relative epilimnion abundances in March, April, and May (Fig. 3A). Each of the four algaviruses (0001, 0002, 0003, and 0008) exhibited a relative abundance >5.3% for at least 1 month and MAG 0001 alone accounted for 47.6% of the relative abundance in March. Among the four viruses, two (0001, 0003) were related to Yellowstone Lake phycodnaviruses of the *Prasinoviridae* (AG_01) family, with ANIs of 87.5% and 84.1%, respectively [46]. The other two (0002, 0008) lacked any close relative in the database. Besides algaviruses predominating in the epilimnion in spring, the relative abundance of a mirusvirus (0007) ranked second and third among all GV MAGs in March (5.4%) and April (6.5%), respectively. Another mirusvirus (0010) ranked fourth in April, accounting for 5.3% of the relative abundance. Additionally, three viruses (0009, 0040, and 0018) with ANIs >87% to the reference genomes of the *Mesomimiviridae* family, and one virus (0014) with an ANI of 77.1% to a reference genome of the *Allomimiviridae* (IM_12) family, were among the most abundant species identified. Each accounted for >2.1% of the relative abundance in the epilimnion during this period. Together, mirusviruses and nucleocytoviruses of the orders *Algavirales* (family *Prasinoviridae*) and *Imitervirales* (families *Mesomimiviridae* and *Allomimiviridae*) were major players in the epilimnion of Lake Biwa in spring.

In addition to the topology of HK97 MCPs, the dynamics of freshwater mirusviruses agreed with the differentiation of three subclades defined by the phylogeny of HK97 MCPs. The community dynamics of subclade1 exhibited a typical hypolimnion-specific pattern (Fig. 4C), occurring only in the hypolimnion throughout the stratified period. In sharp contrast, subclade 2 was transient, occurring exclusively in the epilimnion in April (at the end of water mixing) but absent from both water layers during the stratified period. The members of subclade 3 were typically epilimnion-specific and persistent.

## Discussion

### High-quality freshwater giant virus metagenome-assembled genomes assembled from long reads

Although GVs exhibited high genetic diversity, they accounted for only ~2.0% of all metagenomic reads (Fig. S5), much lower than the value reported for prokaryotes in the same size fraction (0.2–5 μm) of the same samples (60.4%) [16]. This combination of high diversity and low read abundance posed a challenge for reconstructing GV MAGs. Nevertheless, our pipeline (Fig. S3) successfully captured such diversity in Lake Biwa as demonstrated by the inclusion of most viral *polB* genes in the MAGs (Fig. 1C). Although GV MAGs have been widely recovered using short-read sequencing [3–6], no long-read GV MAGs from freshwater lakes have previously been reported. Through comparisons with short-read MAGs, we validated the performance of long reads in terms of the recovery of more continuous and complete GV MAGs (Fig. S6).

Our spatiotemporal sample data focusing on an under-investigated freshwater system expanded the known diversity of GVs, with many new species (97% of all species detected). A large proportion of MAGs had diverged from known reference species, primarily those with ocean-derived genomes [3–6]. Among those related to known GV references, most were closely related to freshwater-derived genomes, suggesting the existence of ecological barriers between the GVs of freshwater and marine environments. Furthermore, we discovered mirusviruses in the freshwater lake.

### A giant viruses community specific to the dark hypolimnion

As observed for prokaryotes [16] and their viruses [47, 48], the GV community followed the physical structure of the seasonally stratified lake water column (Fig. 3B). Most species exhibited clear niche specificity for either the epilimnion or hypolimnion (Figs S14–S16 and 3D). The activities of hypolimnion-specific GVs were characterized by their persistent yet active turnover (Fig. 4B), associated with drastic increases and decreases in abundance over a short period of time. For example, the relative abundance of MAG 0019 increased fourfold from June to July and then declined in August (Fig. 4B). Similar dynamics have been previously observed in both GVs and their viruses (virophages) from hypolimnion samples of another freshwater lake [49]. Together with the observation that the viral host communities (microbial eukaryotes) are also vertically stratified [50], it is highly probable that hypolimnion-specific GVs actively infect hypolimnion-specific hosts. Unfortunately, our attempts to infer the hosts using gene phylogeny and genomic similarities to the isolated viruses were unsuccessful due to the lack of useful reference genomes in the database. Previous studies have revealed the existence of deep-water specific microbiomes including prokaryotes, their viruses, and eukaryotes [7–10, 50–55]. The persistence and exclusive presence of GV populations in the hypolimnion suggests that they are also part of a deep-water specific microbiome, likely playing important ecological roles in dark environments.

In contrast to hypolimnion-specific GVs, epilimnion-specific GVs were more diverse (Fig. 3D) and typically transient (Fig. 4A), in agreement with the observed associations between the GVs and transient hosts including surface bloom-forming algae (Figs 3A and S14) [56, 57]. GVs have been widely studied in the context of algal blooms, especially in the oceans, where they play critical roles in bloom termination [58–60]. However, the major GVs involved in freshwater spring blooms remain largely unknown. A recent study isolated a bloom-associated imitervirus of the *Allomimiviridae* family from a freshwater lake [61], and another work characterized the GV community dynamics associated with algal blooms [11]. Our study revealed that mirusviruses and nucleocytoviruses of the orders *Algavirales* (family *Prasinoviridae*) and *Imitervirales* (families *Mesomimiviridae* and *Allomimiviridae*) were among the most abundant GV lineages during the spring bloom of Lake Biwa. In contrast, members of *Algavirales* did not exhibit a significant presence during the autumn bloom (Fig. 3A). The results suggested that members of *Algavirales* may infect algae of the *Cryptophyceae* and *Chrysophyceae* classes, because they were exclusively dominant during spring bloom (Fig. S17).

A previous study reported high vertical connectivity among GV communities in marine environments that were suggested to be associated with sinking algae [62]. However, our data reveal a disconnect between the epilimnion and hypolimnion GV communities. Algaviruses (0001 and 0003) that dominated in the epilimnion during the spring bloom in May (Fig. 3A) were rarely observed in the hypolimnion during the water stratification period (Fig. S14) despite that significant sinking algal fluxes have been observed in Lake Biwa [63, 64]. Among the algavirus populations in the hypolimnion, an algavirus (0047), different from those dominant in the epilimnion, was the most abundant throughout the lake stratification period (Fig. S16). These results suggest that the transportation of GVs from the epilimnion to hypolimnion associating with algal cells sinking is limited in Lake Biwa.

### Ubiquitous freshwater mirusviruses across water depths

GVs were previously known as large viruses of the phylum *Nucleocytoviricota.* A recent oceanic survey discovered a new GV phylum, *Mirusviricota* [5], which highlighted the importance of linking the evolutionary paths of the two viral realms *Varidnaviria* and *Duplodnaviria* [65]. The cited work reported that mirusviruses were abundant and widespread in sunlit oceanic areas. However, it remained unclear whether mirusviruses existed in freshwater ecosystems and aphotic regions. A recent work reported mirusvirus genomic fossils in the genomes of various eukaryotes including freshwater algae and cellular slime molds of soil, suggesting that their habitats are broad [34]. The recovery of mirusvirus genomes from Lake Biwa has firmly established their existence and activity in freshwater systems. The phylogenetic inference of the marker gene (Fig. 2A) suggests that the freshwater mirusviruses form a distinct lineage that is placed outside the marine clade. The placement of subclade3 within the clade E03 of endogenous mirusvirus HK97 MCPs detected from algae of the class *Cryptophyceae* suggests that these algae may be hosts for mirusviruses of subclade3. The unique gene repertoires (Table S4; Fig. S11) of our freshwater mirusviruses also demonstrate an untapped source of genetic diversity.

We found one circular mirusvirus genome (0074) confined to the dark hypolimnion (Fig. 4C), possibly infecting hypolimnion-specific hosts. A previous oceanic survey of GVs across water columns from surface to 5500 m did not detect any mirusvirus genome below the photic layer [6]. Recent studies have reported that the protist *Aurantiochytrium limacinum* (Labyrinthulea) [66] and green algae *Cymbomonas tetramitiformis* [67] are highly probable hosts of mirusviruses. However, these organisms typically inhabit sunlit regions. Our observation of a hypolimnion-specific mirusvirus highlights the potential roles of mirusviruses as components of the deep-water specific microbiomes of freshwater ecosystems.

### Giant virus rhodopsins in a dark environment

Our results suggest that GV rhodopsins might have light-independent functions as we observed their broad distribution across various habitat preferences and taxonomic groups, including mirusviruses (Fig. S13). The viral type-1 rhodopsins of nucleocytoviruses [68–73] were previously thought to be involved in light absorption and sensing, in turn influencing the behaviors of photosynthetic hosts during infection. HeRs are actively expressed in marine mirusviruses, especially those of sunlit oceans [5]. Initial characterization of HeRs suggested light-sensory activity [74]. However, a recent work has pointed to a different perspective on their roles as all HeRs in theionarchaeal (archaeal) genomes were identified in light-insufficient environments [75]. Our report of rhodopsin genes among hypolimnion-specific GVs suggest previously under-investigated functions in dark habitats.

### Potential high dispersal rates of giant viruses

We recovered three imiterviruses (Fig. S8) from Lake Biwa that were nearly identical (ANIs of 99.2%, 98.2%, and 98.2%) to those of Lake Lanier in North America [4], suggesting a recent dispersal event between two lakes ~11 220 km apart. In terms of microbial dispersal, prokaryotes have been the foci of previous studies. However, a vigorous debate continues regarding whether these microbes are globally distributed at the species level [76–79]. Although GV dispersal has not been well-studied, a few long-distance dispersal events across continents have been suggested [80]. Two mimiviruses with nearly identical genomes (ANI of ~99.9%) have been isolated in Japan [81] and the UK [82]. Two marseilleviruses sharing ~98.5% of ANIs have been isolated in China (GenBank MG827395) and France [83]. Although these observations imply long-distance GV dispersal, a recent study revealed a high degree of endemism among lakes of the two poles and within each polar region [84]. Quantitative studies on the dispersal vs. local diversification rates of GVs will aid further understanding of the processes shaping the global biogeography of GVs. Deep lakes, given their high levels of physical isolation, would serve as useful models for such quantitative studies.

## Conclusion

Using a combination of spatiotemporal sampling and long-read metagenomics, we revealed previously under-investigated diversity of freshwater GVs, as evidenced by 285 new species and the discovery of viruses of the phylum *Mirusviricota* in a freshwater lake. We demonstrated the habitat preferences and community dynamics of GVs. Most nucleocytoviruses and mirusviruses could be clearly classified as being specific to either the epilimnion or hypolimnion. Epilimnion specialists tended to be transient, whereas hypolimnion specialists were typically more persistent. These distinctive dynamic patterns suggest that GVs employ diverse ecological strategies, and our work paves the way towards a better understanding of the roles played by GVs in microbial ecosystems. Specifically, the strong seasonality in the epilimnion suggests that GVs make significant contributions to the plankton community shift in the lake. Not only nucleocytoviruses but also mirusviruses are major players during the spring bloom. Conversely, persistent hypolimnion specialists that nonetheless exhibit active turnover suggest that GVs also play unique and important roles in the hypolimnion-specific microbial ecosystem. Furthermore, our observation of nearly identical GVs in lakes of different continents suggests a ubiquitous distribution of GVs at the species level, highlighting the role of dispersal in shaping the global distributions of GV communities. In light of this, we call for research on GV host interactions, diversity, and biogeography in freshwater lakes worldwide, providing key insights into the eco-evolution of GVs in such unique ecosystems.

## Supplementary Material

Supplementary_Information_wrae182

Supplementary_tables_wrae182

## Data Availability

The nucleotide and protein sequences of the LBGVMAGs, along with alignment and tree files for the phylogenetic analyses conducted in this study, are available at GenomeNet: https://www.genome.jp/ftp/db/community/LBGVMAGs/. Additionally, polished contigs assembled in the previous study, which were used to recruit single-contig GV MAGs in this study, are also accessible at the same location.
