# Supplementary material for: Spatiotemporal dynamics of giant viruses within a deep freshwater lake reveal a distinct dark-water community
Source: ISME J. 2024 Sep 23;18(1):wrae182. doi: 10.1093/ismejo/wrae182 (PMC11465185; doi:10.1093/ismejo/wrae182)
Supplement: Supplementary_Information_wrae182 [file supplementary_information_wrae182.docx]

# **Supplementary Information**

# **
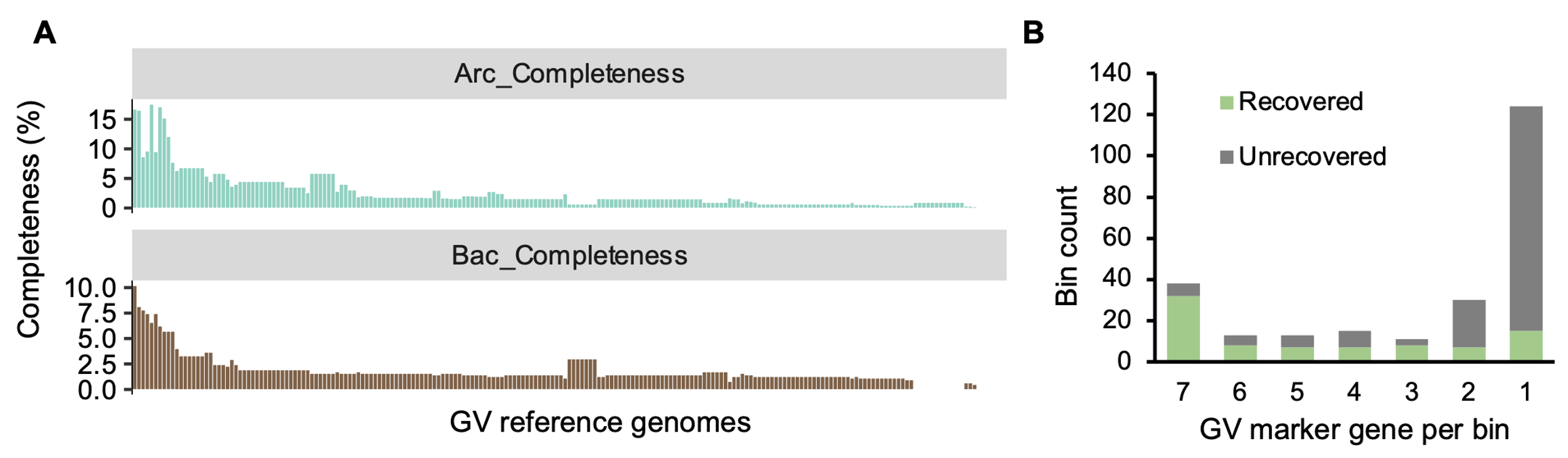
Supplementary Figures**

**Figure S1.** **Evaluation of the exclusion criteria for prokaryotic genomes.** (A) Completeness value of 207 nucleocytovirus reference genomes estimated by CheckM. Each bar represents one genome. The upper panel was estimated using a set of archaeal marker genes, and the lower panel used a set of bacterial marker genes. Based on this result, we used the completeness scores of 20% and 15% as the thresholds to exclude achaeal and bacterial metagenome assembled genomes from our dataset, perspectively. (B) Recovery of giant virus (GV) signatures in the excluded bins by CheckM. The x-axis shows the count of marker genes detected in each excluded bins. Bins with no GV marker genes are not shown here. A bin was considered recovered if its GV signatures were also identified in our final GV MAGs (See the Supplementary Methods).

**
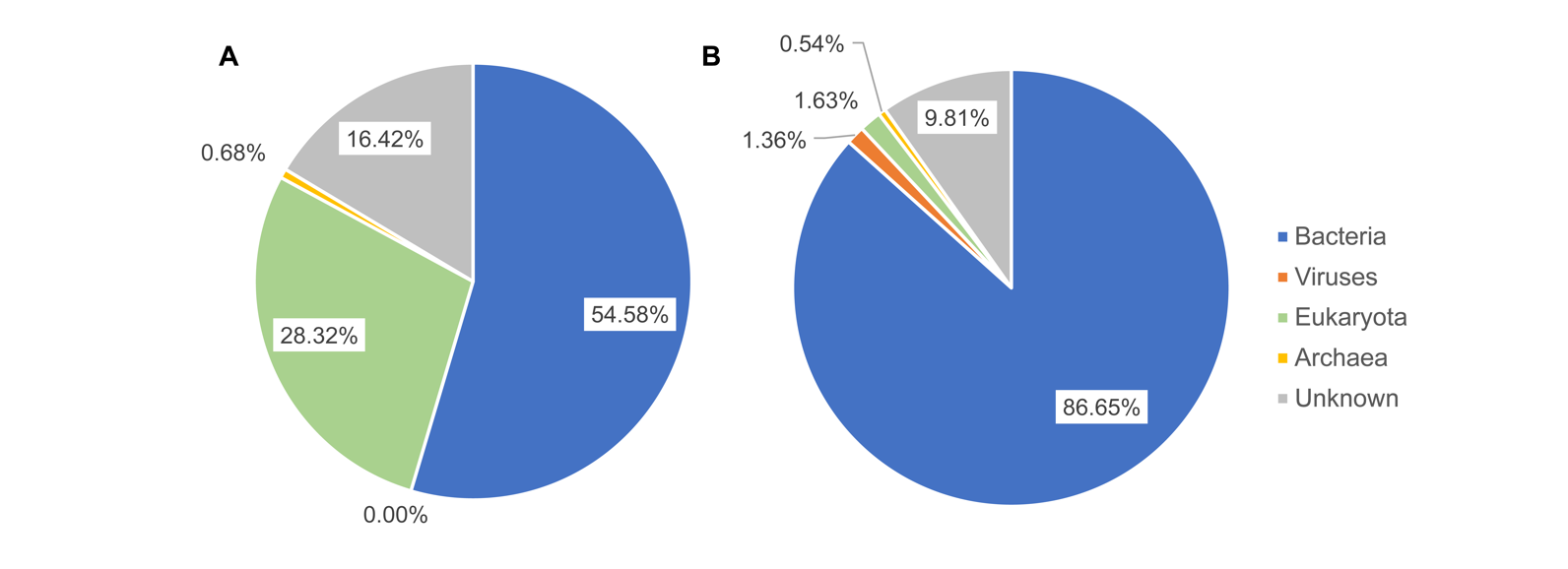
Figure S2. Taxonomic annotation of removed cellular contamination.** The removed cellular contamination includes nonviral contigs (A) and partial cellular regions within chimeric contigs (B) as identified by CheckV (see the Supplementary Methods). The taxonomy of these removed sequences was assigned using CAT to validate the performance of CheckV as plotted.

**
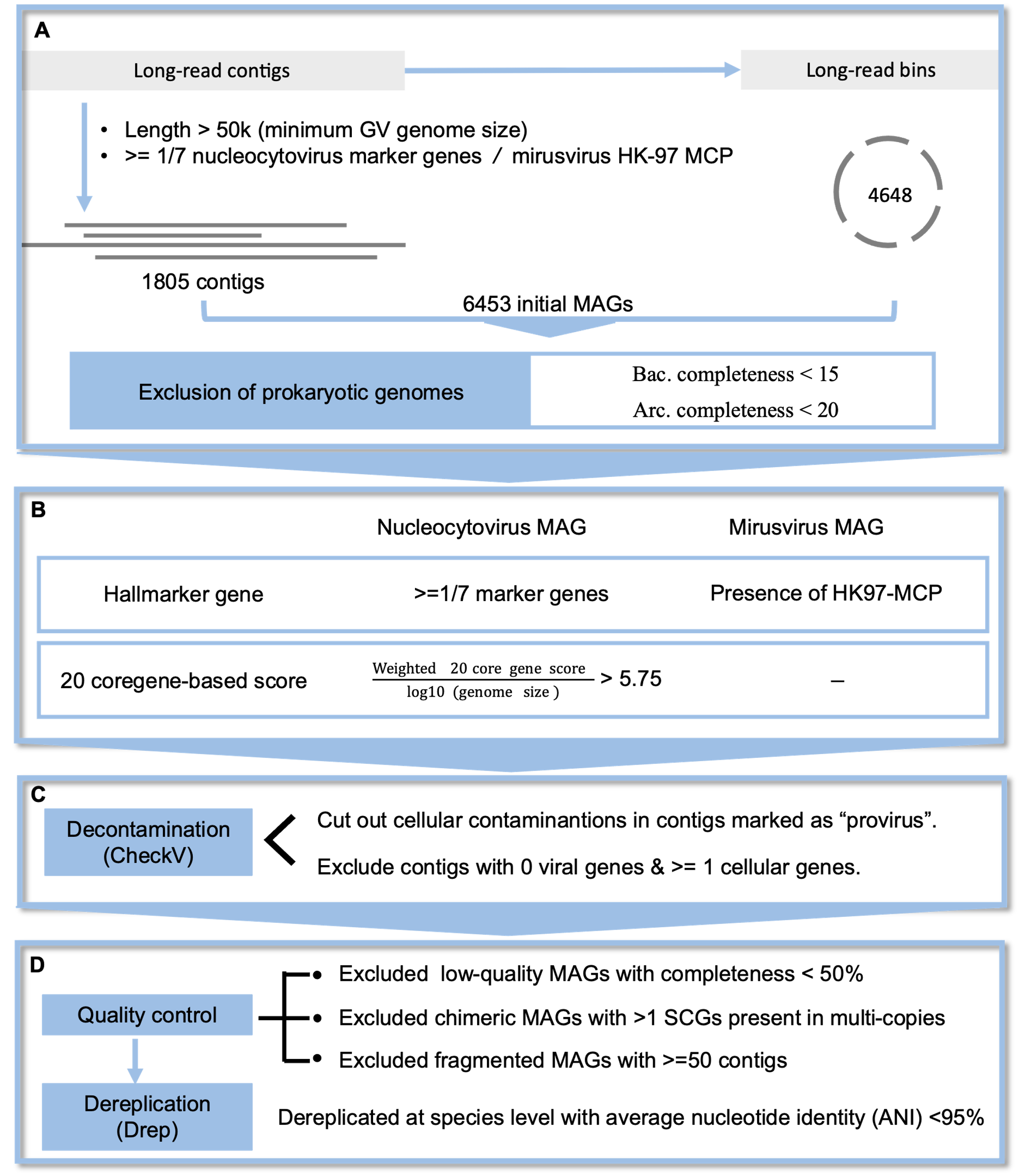
Figure S3.** **Pipeline for recovering long-read GV MAGs**. In this pipeline, we not only recovered bins but also recruited nearly complete GV contigs by taking advantage of long-read sequencing. A customized pipeline guided by HK97 MCP gene was used to recover miruvirus MAGs.

**
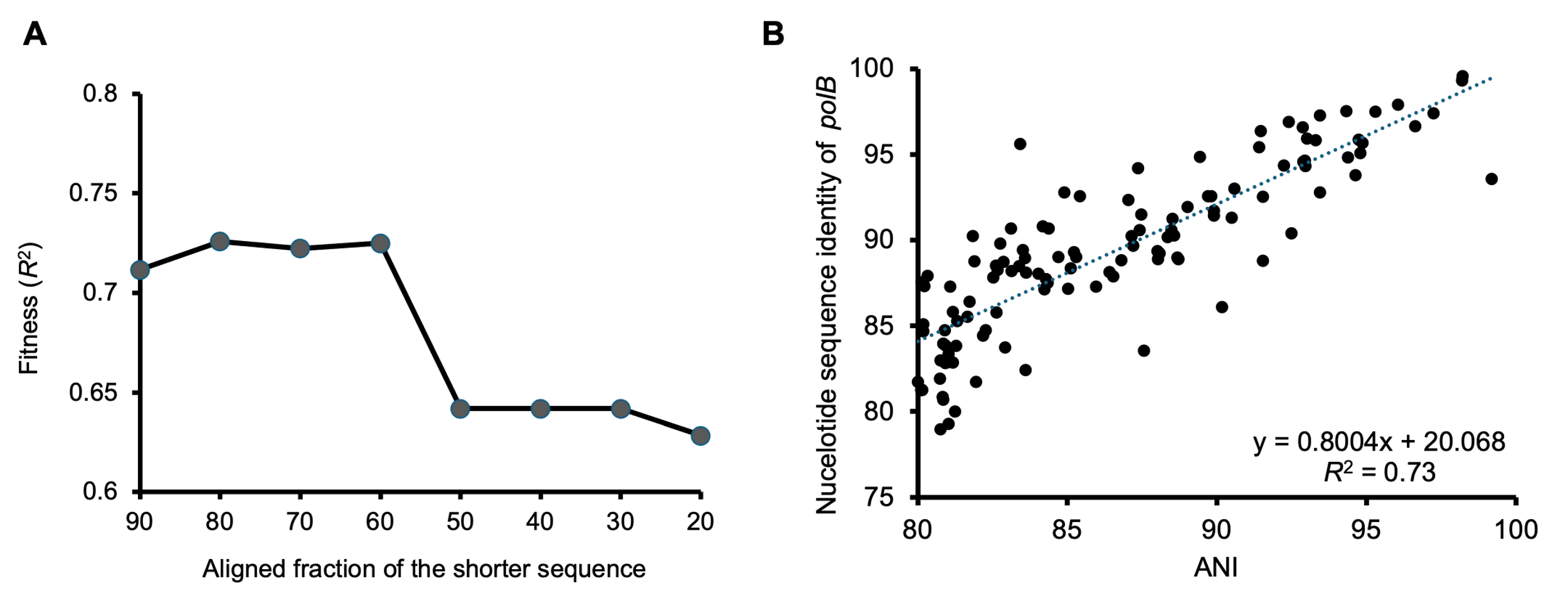
**

**Figure S4. The correlation between pair-wise average nucleotide identity and the *polB* nucleotide sequence identity.** For this analysis, we ultilized two public databases in addition to our GV MAGs (See the Supplementary Methods). To determine the nucleotide sequence identity of *polBs* corresponding to the species boundary of GVs, we correlated pair-wise nucleotide sequence identity of *polB* with average nucleotide identity (ANI) and fitted the trend with a linear trendline. For this fitting, we included only genome pairs with ANIs >80% and tested the fitness (y-axis) across aligned fractions of *polBs* (x-axis) from 90% to 20% (A). We observed a drop in fitness at an aligned fraction of 50%, indicating that fractions below this threshold were not informative enough for correlation. Therefore, we selected pairs with aligned fractions >60% of the shorter sequence in a pair for the correlation. From this, we estimated that an ANI of 95% corresponds to a sequence identity of about 96% in blastn analysis of *polB* sequences (B), representing the species boundary of GVs.

**
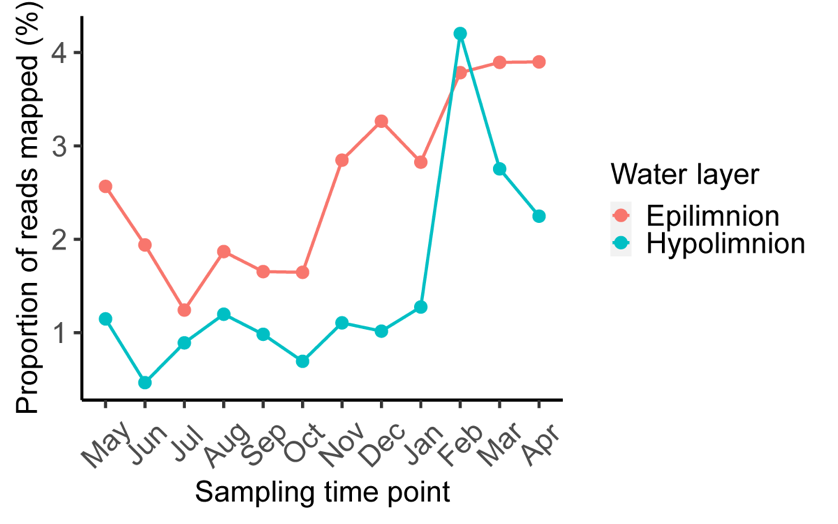
Figure S5. Proportion of short reads mapped onto GV MAGs across 24 samples.** The short reads were mapped to the GV MAGs, and the proportion of mapped reads in each sample was plotted.


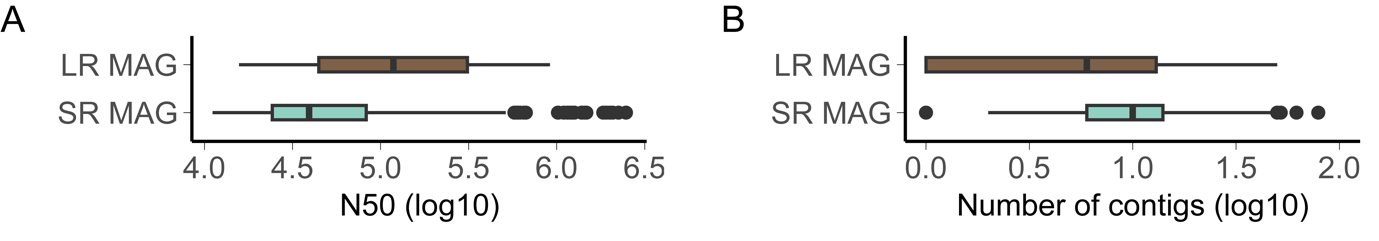
**Figure S6. Comparison between long-read and short-read MAGs.** (A) Comparsion of the N50 (*P* value = 8.4×10^-16^) between our long-read GV MAGs (LR MAG) and high/medium-quality short-read MAGs from GVDB (SR MAG).(B) Comparsion of number of contigs per MAG (*P* value = 6.8×10^-6^).

**
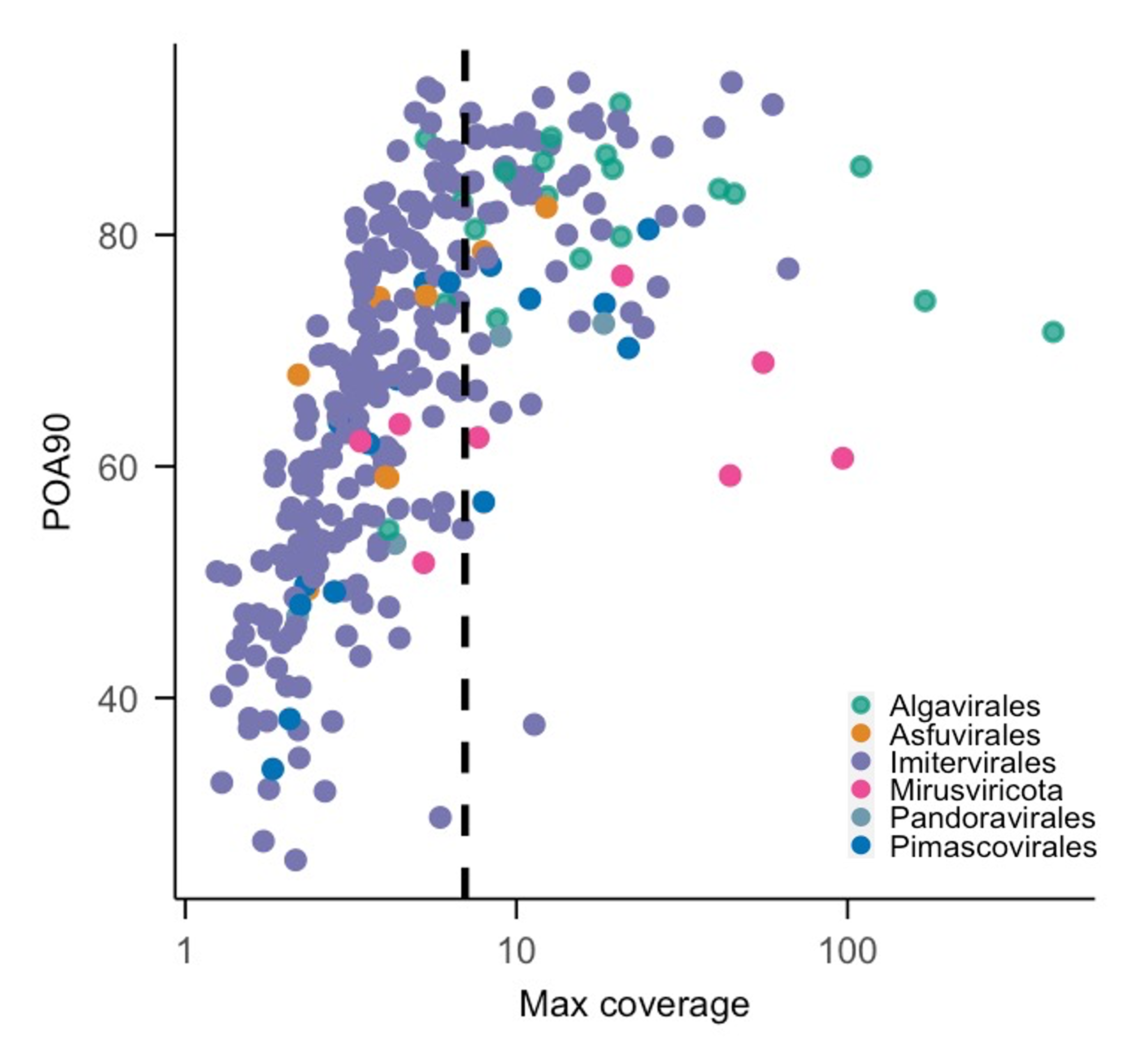
Figure S7.** **POA90 score of GV MAGs.** The dashed line indicated the threshold of coverage (7x), suggesting the indel errors were effectively corrected in the GV MAGs.


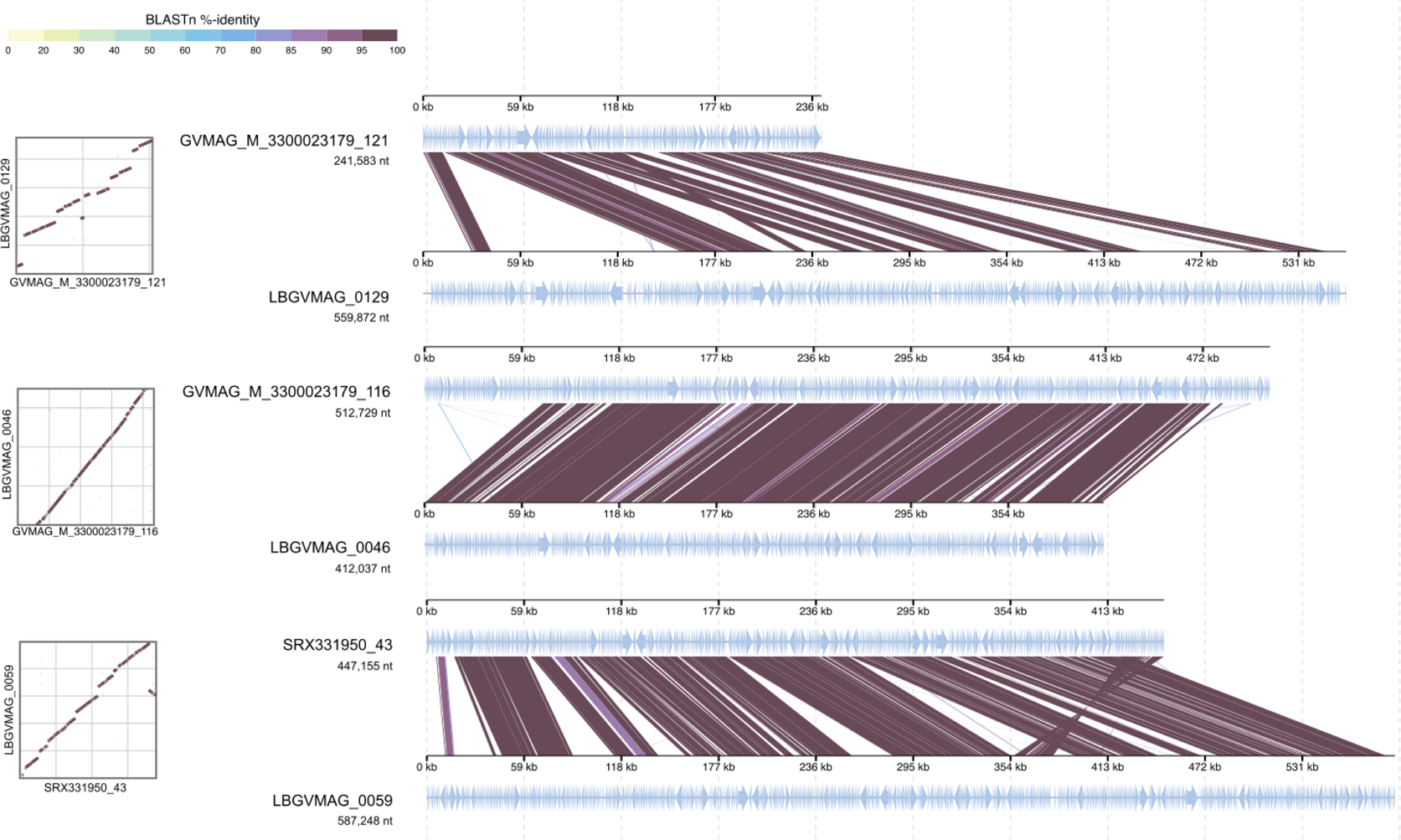
**Figure S8. Blastn alignment of almost identical GV genome pairs.** From the top to bottom, the average nucleotide identities (ANIs) of the pairs were 99.18%, 98.21%, and 98.19%, respectively. Within each pair, the MAG on top was from Lake Lanier, and the bottom was GV MAG recovered in this study from Lake Biwa. Contigs were reordered and concatenated within each genome for better visualization.


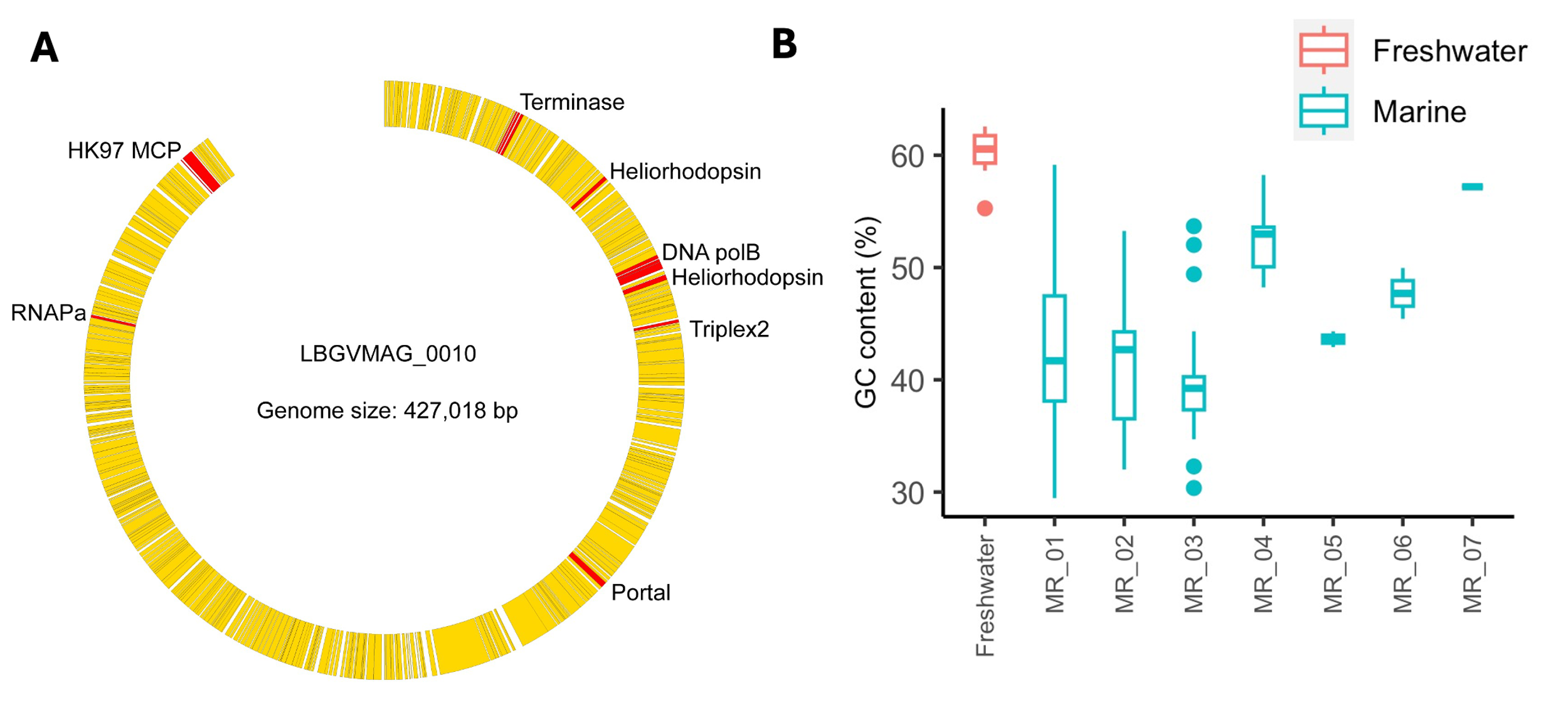
**Figure S9.** **Comparison of genomic features between freshwater and marine mirusviruses.** (A) Key homologues of marine mirusviruses found in a freshwater mirusvirus genome (0010) as an example. The yellow boxes are predicted genes of this genome and key homologues are highlighted in red. (B) Comparison of GC content between freshwater and marine mirusvirus clades (MR_01-07).

**
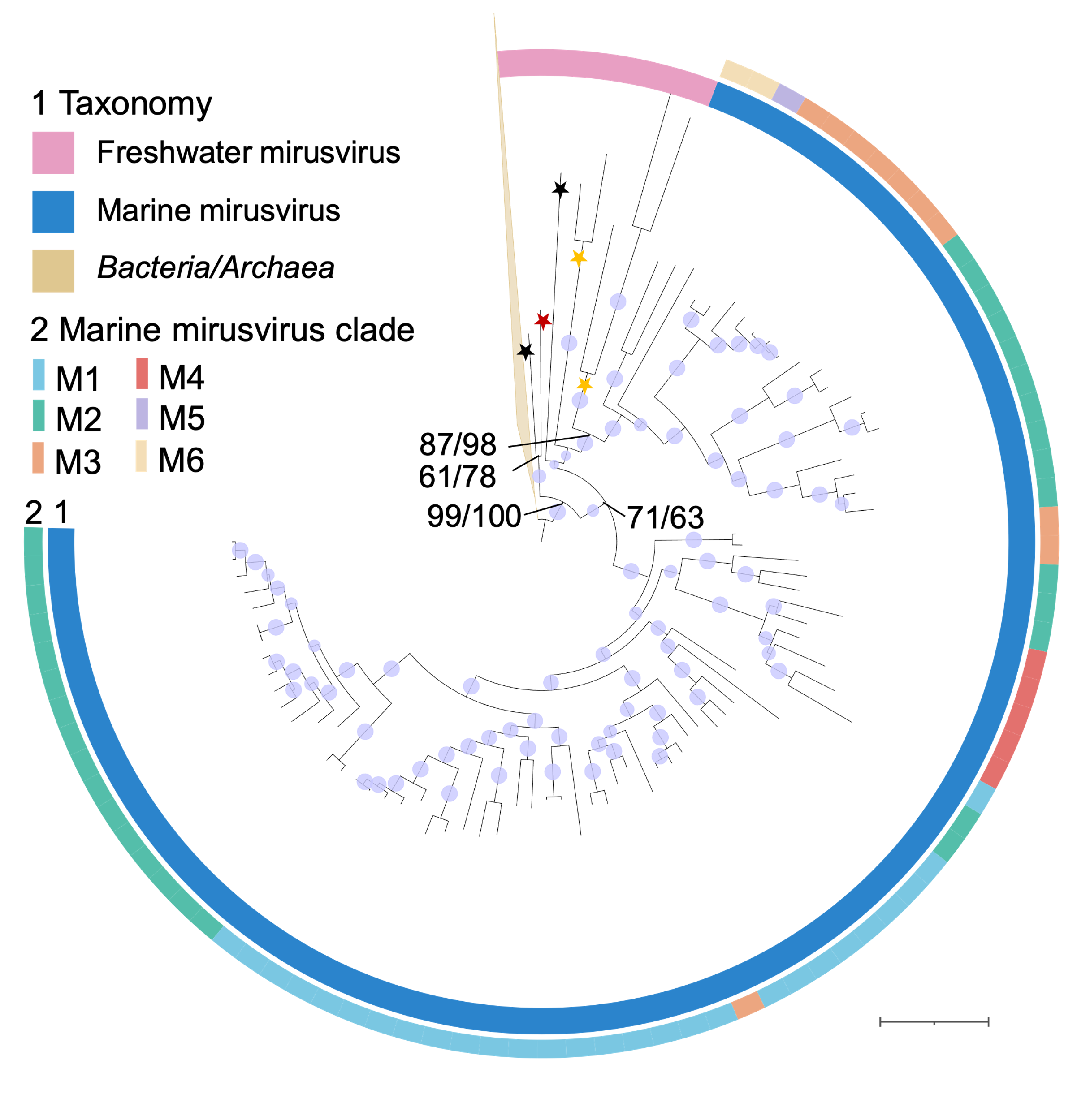
Figure S10.** **Phylogenetic tree of mirusvirus heliorhodopsins**. The tree includes sequences of freshwater mirusviruses, marine mirusviruses and reference sequences of bacteria and archaea downloaded from RefSeq database. The tree was rooted between the clasped branch of bacterial and archaea heliorhodopsin sequences and the rest mirusvirus sequences. Three proposed subclades of freshwater mirusviruses were marked with stars on the tree (subclade1: red; subclade2: black; subclade3: yellow). The Ultrafast Bootstrap value of each node was shown by the size of circles on the nodes. Scale bar represents one substitution per site. Certain key nodes indicating the divergence of different clades were specified by Ultrafast Bootstrap values (aLRT/UFBoot) (see the Methods). Phylogenetic supports were considered high (aLRT>=80 and UFBoot>=95), medium (aLRT>=80 or UFBoot>=95) or low (aLRT<80 and UFBoot<95).

**
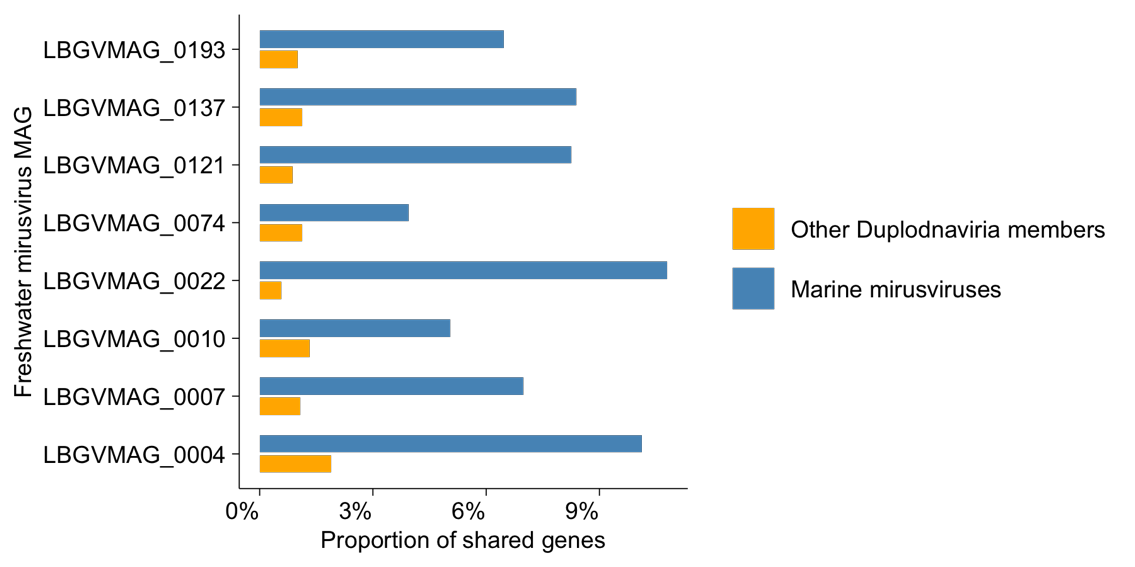
Figure S11. Proportion of shared genes per genome between freshwater mirusviruses and two other groups: marine mirusviruses and other members of the realm *Duplodnaviria*.** An orthology group (OG) was considered shared if it was detected from more than one of these groups. The shared proportion was determined by dividing the number of genes in shared OGs by the total number of predicted genes in each genome. Annotations of shared OGs are available in Table S4.

**
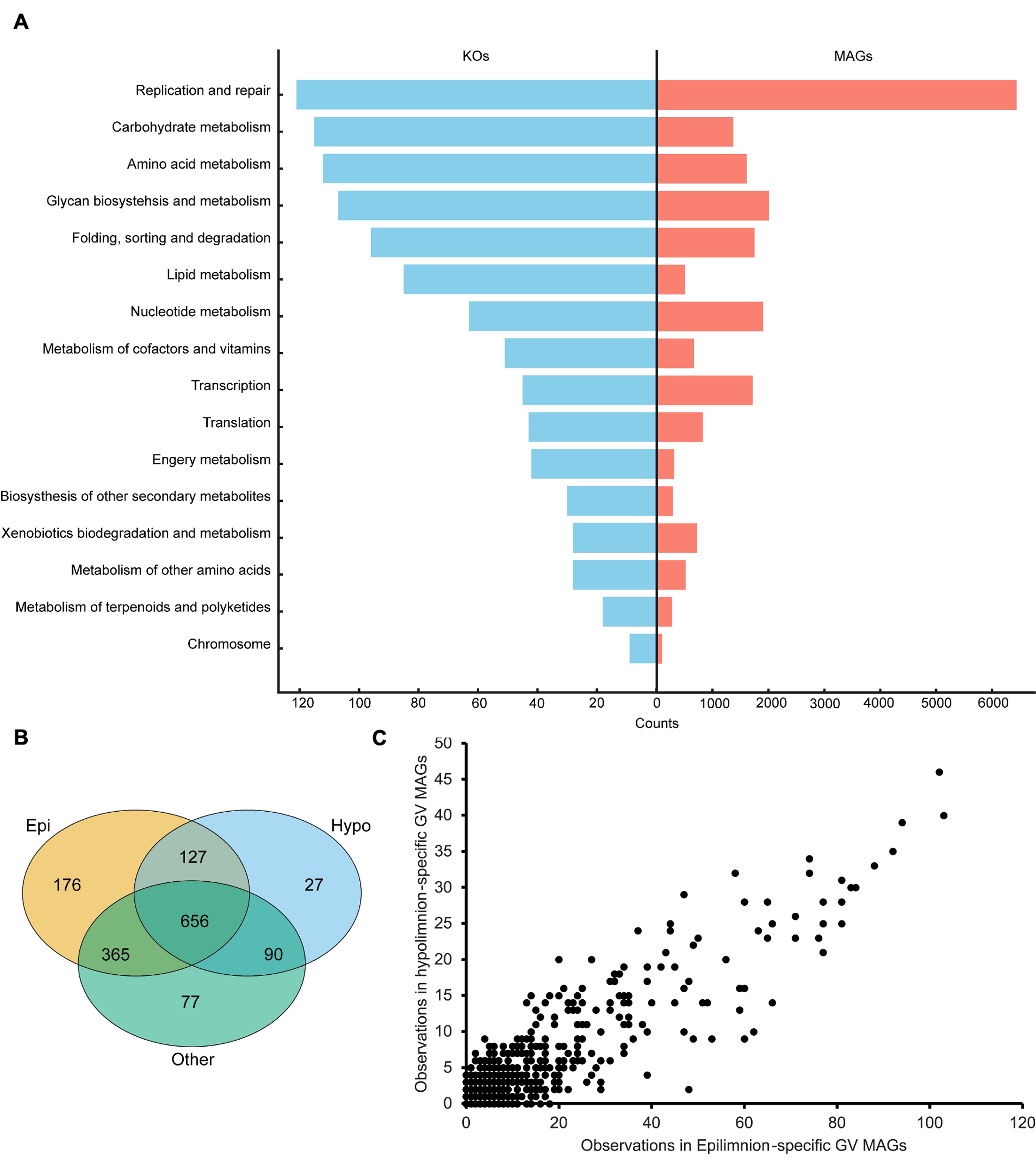
Figure S12. Distributions of KOs and KEGG pathways identified from GV MAGs.** We classified KEGG orthology (KO) identified from GV MAGs into corresponding KEGG pathways. (A) The number of KOs in each pathway is shown in the left panel and the accumulative count of GV MAGs, from which KOs involved in each pathway were detected, is shown in the right panel. Among these pathways, those involved in genetic information processing include “replication and repair”, “folding, sorting and degradation”, “transcription”, “translation”, and “chromosome”. (B) Venn diagram showing the number of KOs detected in MAGs specific to epilimnion (Epi), hypolimnion (Hypo), and MAGs without a habitat preference (Other). (C) Genome count of each KO by habitat preference. Each dot represents one KO. The x-axis and y-axis denote the number of epilimnion- and hypolimnion-sepcific GV MAGs from which the KO was detected, respectively.

**
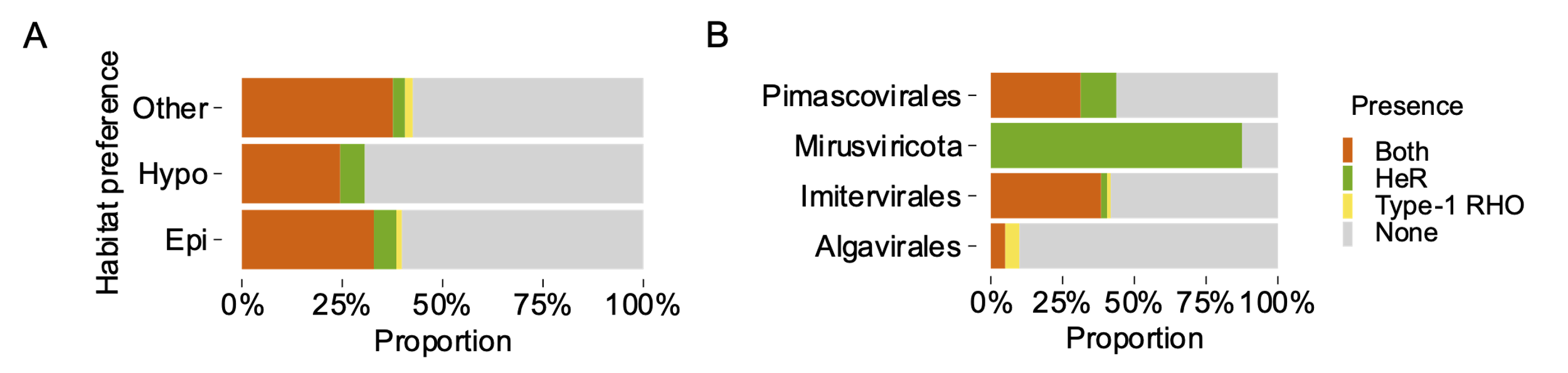
Figure S13. Microbial rhodopsin-encoding gene detected from GV MAGs.** In both figures, we focused on the presence or absence of rhodopsin genes in the MAGs. Multiple copies of a type-1 rhodopsin (Type-1 RHO) or heliorhodopsin gene (HeR) within a MAG were treated equivalently to single copy, and thus marked as 'present'. The proportion of MAGs with rhodopsin genes present was calculated as the percentage of all GV MAGs in which rhodopsins were identified.


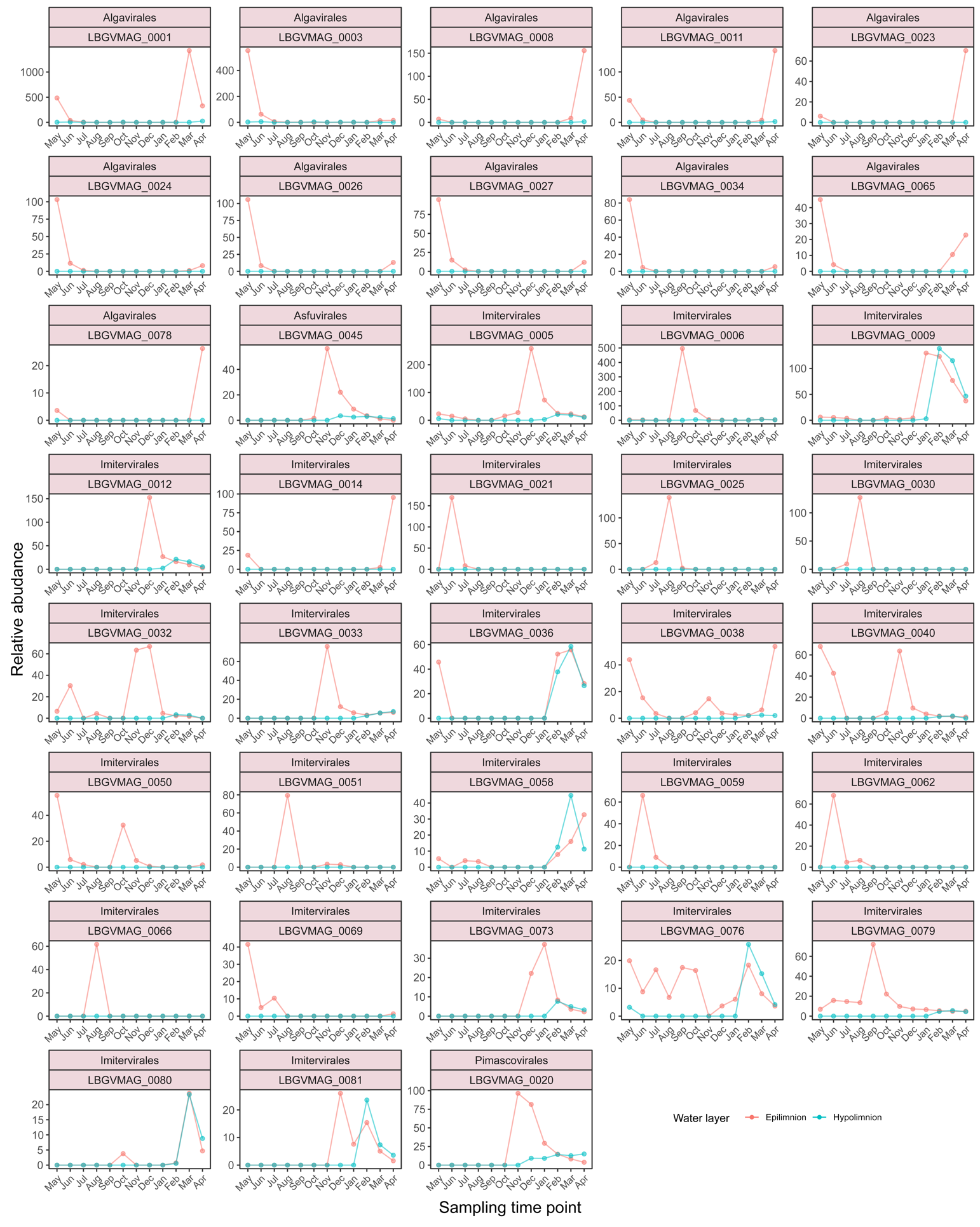
**Figure S14. Community dynamics of epilimnion-specific nucleocytoviruses.** Epilimnion-specific MAGs with coverages >7× were selected for this analysis based on relative abundance (RPKM). The title of each box included taxonomy (the first line) and MAG ID (the second line).

**
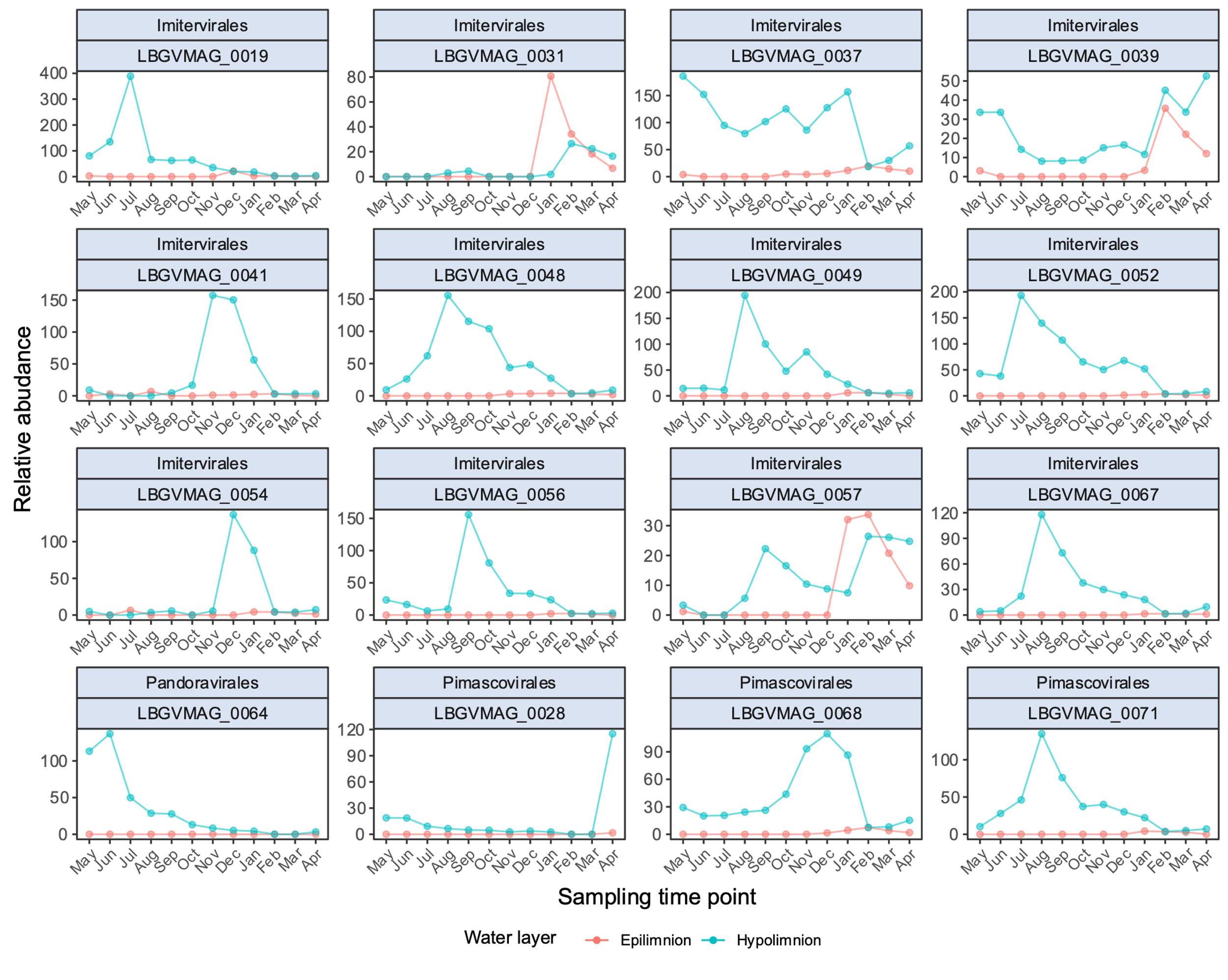

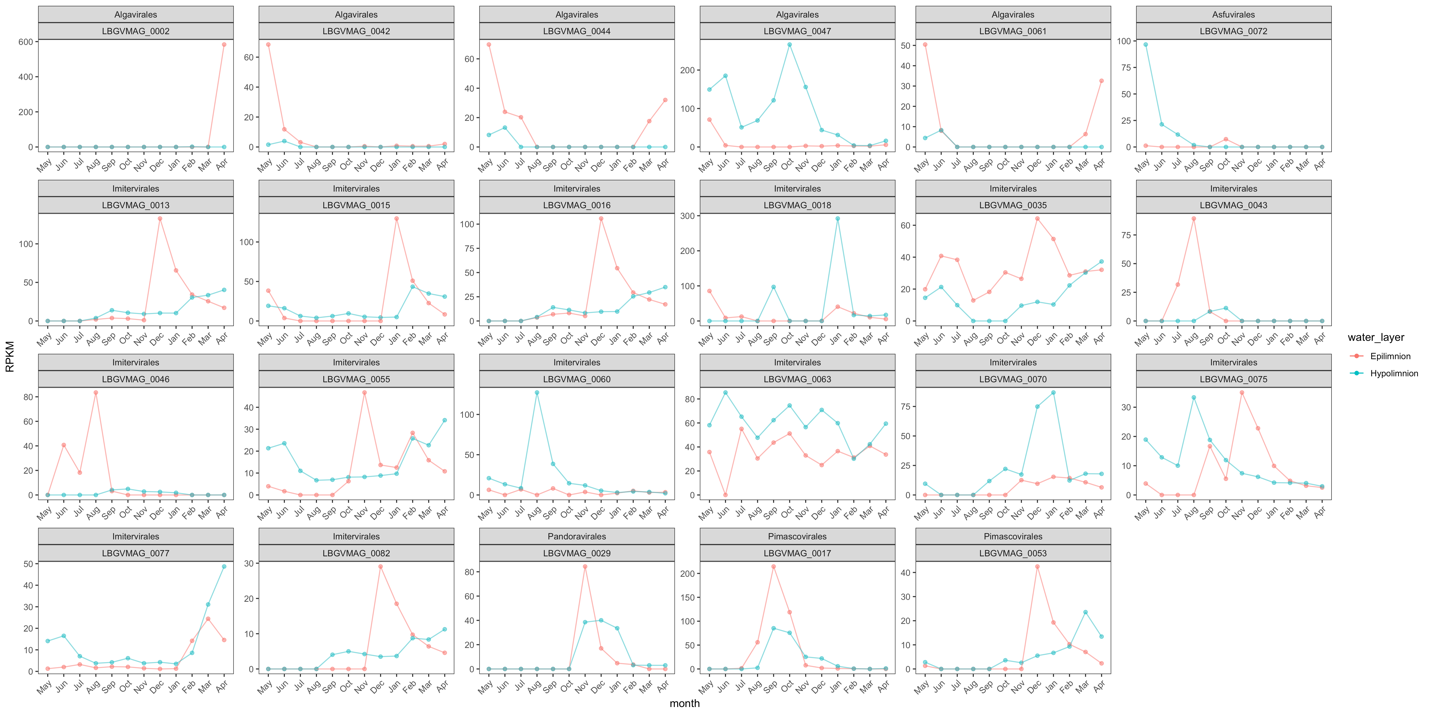
Figure S15. Community dynamics of hypolimnion-specific nucleocytoviruses.** Those hypolimnion-specific MAGs with coverages > 7× were selected for this analysis based on relative abundance (RPKM). The title of each box included taxonomy (the first line) and MAG ID (the second line).

**
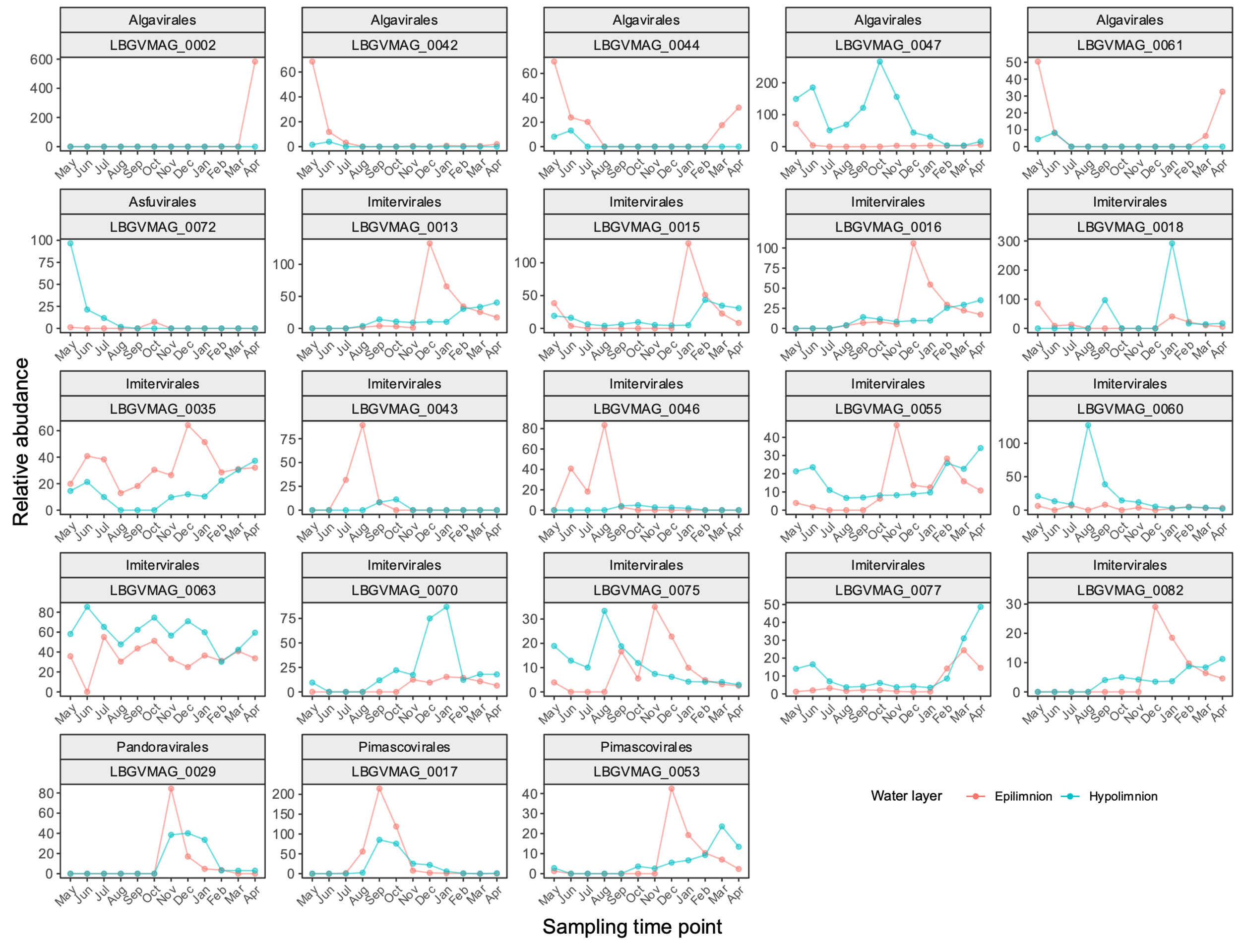
Figure S16. Community dynamics of nucleocytoviruses with no identified habitat preference.** Those vertically ubiquitous MAGs with coverages > 7× were selected for this analysis based on relative abundance (RPKM). The title of each box included taxonomy (the first line) and MAG ID (the second line).


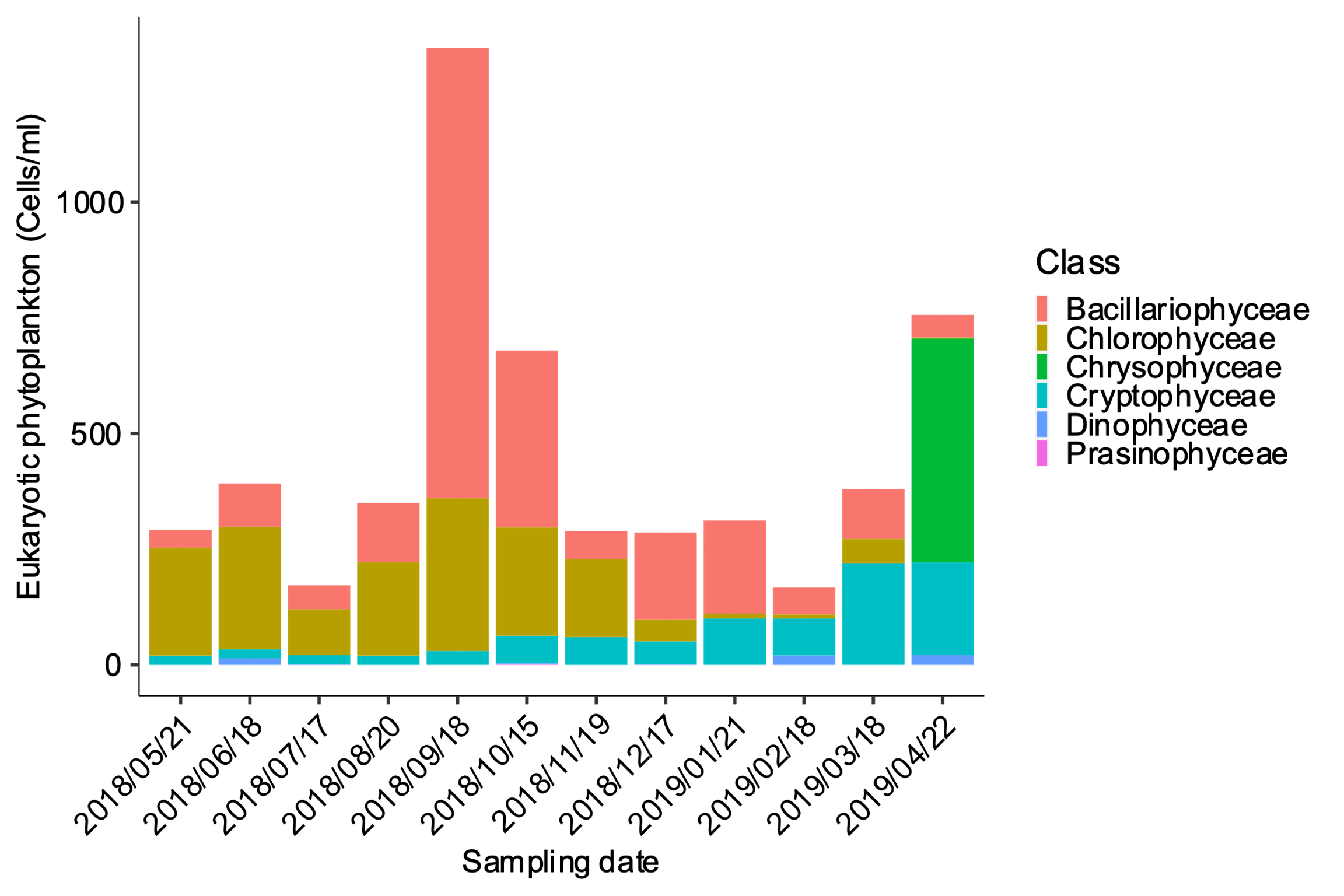
**Figure S17. Phytoplankton dynamics in the north basin of Lake Biwa.** This data was downloaded from a Lake Biwa monitoring project (see the Methods), with water samples collected at a depth of 0.5 meters. Cyanobacteria are omitted from this figure as they fall outside the host range of GVs.

**Legends for Supplementary Tables**

**Supplementary Table 1. Description of the 293 LBGVMAGs.** This table includes basic descriptions of the 293 GV MAGs recovered in this study, including the genome size, number of contigs, N50, habitat preference, number of each marker gene detected, and abundance profiles.

**Supplementary Table 2. Terminal inverted repeats of GV MAGs.** This table lists six putative terminal inverted repeats detected from GV MAGs, including the length of flanking region by one end of the MAG.

**Supplementary Table 3. ANI values calculated for GV MAGs.** Pair-wise ANI comparisons were done for each GV MAGs against the custom database in this study (See the Methods). only the pair with the highest ANI value for each GV MAG was selected and plotted, thereby highlighting the closest genomic relationships with GV genomes from other environments. The sources of the GV genomes from the two databases are also specified in this table. GV MAGs without specific ANI values, due to large divergence from GV genomes in the custom database, are not included in this table.

**Supplementary Table 4. Shared OGs among freshwater mirusviruses, marine mirusviruses, and other members of *Duplodnaviria*.** This table lists OGs shared among freshwater/marine-originated mirusviruses and other members of *Duplodnaviria*. Annotations of these OGs using three public databases (See the Supplementary Methods) are included.

**Supplementary Table 5. List of KOs detected from GV MAGs.** This table lists KOs annotated from GV MAGs by eggNOG-mapper. Those genes without an assigned KO are omitted from this table.

**Supplementary Methods**

## **Exclusion of prokaryote-like MAGs**

As GVs accounted for a minor proportion of the assembled contigs and bins compared to prokaryotes, we excluded prokaryotic sequences before downstream screening. We employed CheckM v1.2.2 [1] to estimate the completeness of each GV MAG candidate as a bacterial or archaeal genome. To minimize false positives, we tested the threshold for the completeness score to exclude a GV MAG using 207 reference GV genomes [2] (Fig. S1A). A completeness score of 15 for bacteria and 20 for archaea were determined as balanced thresholds to exclude prokaryote-like genomes. GV MAG candidates with completeness scores higher than either of these two thresholds were excluded.

We reevaluated the 244 bins that were excluded by our CheckM filter but contained at least one of the seven GV marker genes. Using blastn, we compared the 244 bins against our final GV MAGs. A contig was considered recovered if its alignment identity was greater than 90% and it covered more than 90% of the length of the shorter contig aligned to our final GV MAGs. Furthermore, the GV signatures in a bin was considered recovered if at least one GV contig was recovered. This reevaluation revealed that GV signatures in 90/244 of the initially excluded bins (by CheckM) were in fact retained in the final GV MAGs (Fig. S1B). The excluded bins typically contained only one or two marker genes, suggesting fragmented GV signals. These fragmented GV signatures have a low recovery rate. In contrast, bins containing six or seven GV marker genes exhibited a higher recovery rate of 84.2% in our refined GV MAGs due to the additional recruitment of single-contig GV MAGs in addition to those of bins (Fig. S3). The majority of these high-quality GV sequences that were mis-binned with prokaryotic genomes were rescued by our original pipeline.

## **Screening for GV MAGs**

For nucleocytoviruses, the core gene density index was determined from the presence of 20 core genes [2]. Gene predictions of the GV MAG candidates were done using Prodigal v2.6.3 with “-meta” mode [3]. Sequences of these 20 core genes, universal or nearly universal in all *Nucleocytoviricota* lineages, were downloaded from the Nucleo-Cytoplasmic Virus Orthologous Groups (NCVOGs) database [4]. Multiple alignments for each NCVOG were performed using MAFFT v7.505, then the corresponding hidden Markov model (HMM) was built with the function ‘hmmbuild’ of HMMER3 v3.4 [5] and used for calculating a core gene density index to screen for putative nucleocytovirus MAGs [2]. Then, we again curated the resulting MAGs by selecting those with at least one of the seven marker genes described above [6]. Occurrences of the seven marker genes were determined using the tool “ncldv_markersearch” [7].

For the detection of mirusviruses, we screened for the mirusvirus marker gene, HK97 MCP gene, in the MAGs. Protein sequences of the HK97 MCP gene from marine mirusvirus MAGs published recently [8] were collected for generating the HMM model as described above, which was used for screening for mirusvirus MAGs.

## **Removal of cellular contamination from GV MAGs**

Since we recovered GV MAGs from the same size fraction (0.2–5 μm) as prokaryotes, mis-binning of a prokaryotic contig was the major source of contamination. Therefore, we further removed prokaryotic sequences from the remaining GV MAGs by screening each contig with “end_to_end” pipeline of CheckV v1.0.1 (database v1.4) [9]. When CheckV detected at least one microbial gene but no viral-specific gene from a contig, the contig was regarded as prokaryotic and consequently removed from the MAG. Through this process, we further found a few contigs annotated as “provirus” by CheckV. Manual inspections indicated that these contigs were not *bona fide* proviruses (GV genomes endogenized in the host genome) but likely resulted from mis-assembly of GV and prokaryotic genomes. For such contigs, we cut out the segments assigned as “host region”, which were defined as non-viral due to the dominance of cellular signals by CheckV. For all regions and contigs removed through this process, we additionally performed taxonomic annotations using CAT [10] to ensure that the removed regions and contigs were indeed contamination (Fig. S2). In this way, we thoroughly removed cellular contamination from the GV MAGs.

## **Exclusion of chimeric, low-quality, and fragmented GV MAGs**

We removed chimeric MAGs in which more than one single-copy marker gene was found in multiple copies [7]. We also excluded fragmented MAGs with more than 50 contigs from further analysis [11]. We removed low-quality MAGs that showed the completeness score lower than 50 [12] using CheckV v1.0.1 [9]. In addition to CheckV, we also identified putative terminal inverted repeats through inverted alignment by Minimap2 v2.26 [13]. We then filtered the alignment result with minimum length of 99 bp and minimum sequence identity of 0.9. Only if the inverted sequences were aligned perfectly from at least one end of the genome and the length of the other flanking end was shorter than the length of aligned sequence, we considered it a terminal inverted repeat. In the case of multi-contig MAGs, we concatenated all the contigs into a single sequence and subsequently performed CheckV on the concatenated contig as CheckV only accepts single-contig input.

## **Dereplication of GV MAGs**

The redundancy of GV MAGs generated from the above processes has been removed based on their average nucleotide identity (ANI) using dRep v.3.2.2 with “--S_algorithm ANImf --clusterAlg single -sa 0.95” parameters. Also, the completeness and contamination scores generated by CheckV v1.0.1 was given by “–genomeInfo” parameter [14]. ANImf with a minimal ANI threshold of 95% was chosen for the secondary clustering because 95% ANI is a commonly used species boundary for GVs [6].

## **Assessment of caputered diversity by recovering *polBs***

We called *polBs* from all the assembled contigs following a pipeline proposed in a previous study [15]. Only *polB* sequences with >500 codons and >7× contig read coverage were used for the analysis. We clustered *polB* nucleotide sequences with cd-hit v4.8.1 [16] at 96% identity and performed the alignment against contigs in GV MAGs.

The threshold of nucleotide sequence identity and the minimum aligned length was determined utilizing two public databases: the Giant Virus Database (GVDB) [6] and 697 nucleocytovirus/mirusvirus MAGs recovered from Tara Oceans, lodged in the Global Ocean Eukaryotic Viral database (GOEV) [8]. We first calculated the pair-wise ANI among our GV MAGs and these two databases using fastANI v1.33 [17]. The polB sequences were called using the tool “ncldv_markersearch” [7]. Then, we correlated the pair-wise nucleotide sequence identity of *polBs* against the pair-wise ANIs and fitted the trend with a linear trendline.

## **Functional annotation**

We assigned KOs to the genes of our GV MAGs with eggNOG-mapper v2.1.12 [18,19]. We excluded genes that only appeared in one GV MAG to increase the confidence of identification. In addition, we also identified rhodopsins by hmmsearch program of HMMER v3.4 against the Pfam model for type1 rhodopsins (PF01036) with a threshold E-value of 1×10^-3^.

## **Indel error assessment of long-read GV MAGs**

The indel errors are common in long-read assembly [20] due to the higher nucleotide error rate than the short-read platforms. Even though the contigs have already been error-corrected (polished) using the long and short read mapping, some errors remained inevitably. Following the previous study [21], POA90 score was defined as the proportion of amino acid sequences in which >90% of the length was aligned to a UniRef90 sequence.

## **Analysis of shared OGs among freshwater/marine mirusviruses and other *Duplodnaviria* members**

We downloaded all available genomes of other *Duplodnaviria* members (i.e., caudoviruses and herpesviruses) from Virus-Host Database [22]. OGs were generated from genomes of three groups (marine/freshwater-originated mirusviruses and other *Duplodnaviria* members) by OrthoFinder v2.5.5 with default parameters [23]. An OG was retained only if it included genes from more than five genomes. Annotations of OGs were done based on Pfam [24], PDB [25], and SCOP70 database [26] by hhsuite v3.3.0 [27].

**References**

1. Parks DH, Imelfort M, Skennerton CT, Hugenholtz P, Tyson GW*.* CheckM: assessing the quality of microbial genomes recovered from isolates, single cells, and metagenomes. *Genome Res* 2015;**25**:1043–55.

2. Fang Y, Meng L, Xia J, Gotoh Y, Hayashi T, Nagasaki K et al. Genome-resolved year-round dynamics reveal a broad range of giant virus microdiversity. [Preprint]. *BioRixv* 2024. DOI: https://doi.org/10.1101/2024.07.08.602415

3. Hyatt D, Chen G-L, LoCascio PF, Land ML, Larimer FW, Hauser LJ*.* Prodigal: prokaryotic gene recognition and translation initiation site identification. *BMC Bioinformatics* 2010;**11**:119.

4. Yutin N, Wolf YI, Raoult D, Koonin EV*.* Eukaryotic large nucleo-cytoplasmic DNA viruses: Clusters of orthologous genes and reconstruction of viral genome evolution. *Virology Journal* 2009;**6**:223.

5. Mistry J, Finn RD, Eddy SR, Bateman A, Punta M*.* Challenges in homology search: HMMER3 and convergent evolution of coiled-coil regions. *Nucleic Acids Research* 2013;**41**:e121.

6. Aylward FO, Moniruzzaman M, Ha AD, Koonin EV*.* A phylogenomic framework for charting the diversity and evolution of giant viruses. *PLOS Biology* 2021;**19**:e3001430.

7. Moniruzzaman M, Martinez-Gutierrez CA, Weinheimer AR, Aylward FO*.* Dynamic genome evolution and complex virocell metabolism of globally-distributed giant viruses. *Nat Commun* 2020;**11**:1710.

8. Gaïa M, Meng L, Pelletier E, Forterre P, Vanni C, Fernandez-Guerra A *et al.* Mirusviruses link herpesviruses to giant viruses. *Nature* 2023;**616**:783–9.

9. Nayfach S, Camargo AP, Schulz F, Eloe-Fadrosh E, Roux S, Kyrpides NC*.* CheckV assesses the quality and completeness of metagenome-assembled viral genomes. *Nat Biotechnol* 2021;**39**:578–85.

10. von Meijenfeldt FAB, Arkhipova K, Cambuy DD, Coutinho FH, Dutilh BE*.* Robust taxonomic classification of uncharted microbial sequences and bins with CAT and BAT. *Genome Biology* 2019;**20**:217.

11. Schulz F, Roux S, Paez-Espino D, Jungbluth S, Walsh DA, Denef VJ *et al.* Giant virus diversity and host interactions through global metagenomics. *Nature* 2020;**578**:432–6.

12. Roux S, Adriaenssens EM, Dutilh BE, Koonin EV, Kropinski AM, Krupovic M *et al.* Minimum Information about an Uncultivated Virus Genome (MIUViG). *Nat Biotechnol* 2019;**37**:29–37.

13. Li H. New strategies to improve minimap2 alignment accuracy. *Bioinformatics* 2021;**37**:4572–4.

14. Olm MR, Brown CT, Brooks B, Banfield JF*.* dRep: a tool for fast and accurate genomic comparisons that enables improved genome recovery from metagenomes through de-replication. *ISME J* 2017;**11**:2864–8.

15. Endo H, Blanc-Mathieu R, Li Y, Salazar G, Henry N, Labadie K *et al.* Biogeography of marine giant viruses reveals their interplay with eukaryotes and ecological functions. *Nat Ecol Evol* 2020;**4**:1639–49.

16. Knowles B, Silveira CB, Bailey BA, Barott K, Cantu VA, Cobián-Güemes AG *et al.* Lytic to temperate switching of viral communities. *Nature* 2016;**531**:466–70.

17. Jain C, Rodriguez-R LM, Phillippy AM, Konstantinidis KT, Aluru S. High throughput ANI analysis of 90K prokaryotic genomes reveals clear species boundaries. *Nat Commun* 2018;**9**:5114.

18. Cantalapiedra CP, Hernández-Plaza A, Letunic I, Bork P, Huerta-Cepas J*.* eggNOG-mapper v2: Functional Annotation, Orthology Assignments, and Domain Prediction at the Metagenomic Scale. *Molecular Biology and Evolution* 2021;**38**:5825–9.

19. Huerta-Cepas J, Szklarczyk D, Heller D, Hernández-Plaza A, Forslund SK, Cook H *et al.* eggNOG 5.0: a hierarchical, functionally and phylogenetically annotated orthology resource based on 5090 organisms and 2502 viruses. *Nucleic Acids Research* 2019;**47**:D309–14.

20. Jain M, Koren S, Miga KH, Quick J, Rand AC, Sasani TA *et al.* Nanopore sequencing and assembly of a human genome with ultra-long reads. *Nat Biotechnol* 2018;**36**:338–45.

21. Okazaki Y, Nakano S, Toyoda A, Tamaki H*.* Long-Read-Resolved, Ecosystem-Wide Exploration of Nucleotide and Structural Microdiversity of Lake Bacterioplankton Genomes. *mSystems* 2022;**7**:e00433-22.

22. Mihara T, Nishimura Y, Shimizu Y, Nishiyama H, Yoshikawa G, Uehara H *et al.* Linking Virus Genomes with Host Taxonomy. *Viruses* 2016;**8**:66.

23. Emms DM, Kelly S. OrthoFinder: solving fundamental biases in whole genome comparisons dramatically improves orthogroup inference accuracy. *Genome Biology* 2015;**16**:157.

24. Mistry J, Chuguransky S, Williams L, Qureshi M, Salazar GA, Sonnhammer ELL *et al.* Pfam: The protein families database in 2021. *Nucleic Acids Research* 2021;**49**:D412–9.

25. Berman HM, Westbrook J, Feng Z, Gilliland G, Bhat TN, Weissig H *et al.* The Protein Data Bank. *Nucleic Acids Research* 2000;**28**:235–42.

26. Lo Conte L, Ailey B, Hubbard TJP, Brenner SE, Murzin AG, Chothia C*.* SCOP: a Structural Classification of Proteins database. *Nucleic Acids Res* 2000;**28**:257–9.

27. Steinegger M, Meier M, Mirdita M, Vöhringer H, Haunsberger SJ, Söding J*.* HH-suite3 for fast remote homology detection and deep protein annotation. *BMC Bioinformatics* 2019;**20**:473.
